# Active photosynthetic inhibition mediated by MPK3/MPK6 is critical to effector-triggered immunity

**DOI:** 10.1371/journal.pbio.2004122

**Published:** 2018-05-03

**Authors:** Jianbin Su, Liuyi Yang, Qiankun Zhu, Hongjiao Wu, Yi He, Yidong Liu, Juan Xu, Dean Jiang, Shuqun Zhang

**Affiliations:** 1 Key Laboratory of Plant Physiology and Biochemistry, College of Life Sciences, Zhejiang University, Hangzhou, Zhejiang, China; 2 Division of Biochemistry, Interdisciplinary Plant Group, and Bond Life Sciences Center, University of Missouri, Columbia, Missouri, United States of America; The Sainsbury Laboratory, United Kingdom of Great Britain and Northern Ireland

## Abstract

Extensive research revealed tremendous details about how plants sense pathogen effectors during effector-triggered immunity (ETI). However, less is known about downstream signaling events. In this report, we demonstrate that prolonged activation of MPK3 and MPK6, two *Arabidopsis* pathogen-responsive mitogen-activated protein kinases (MPKs), is essential to ETI mediated by both coiled coil-nucleotide binding site-leucine rich repeats (CNLs) and toll/interleukin-1 receptor nucleotide binding site-leucine rich repeats (TNLs) types of R proteins. MPK3/MPK6 activation rapidly alters the expression of photosynthesis-related genes and inhibits photosynthesis, which promotes the accumulation of superoxide (${\text{O}}_{2}^{•-}$) and hydrogen peroxide (H_2_O_2_), two major reactive oxygen species (ROS), in chloroplasts under light. In the chemical-genetically rescued *mpk3 mpk6* double mutants, ETI-induced photosynthetic inhibition and chloroplastic ROS accumulation are compromised, which correlates with delayed hypersensitive response (HR) cell death and compromised resistance. Furthermore, protection of chloroplasts by expressing a plastid-targeted cyanobacterial flavodoxin (pFLD) delays photosynthetic inhibition and compromises ETI. Collectively, this study highlights a critical role of MPK3/MPK6 in manipulating plant photosynthetic activities to promote ROS accumulation in chloroplasts and HR cell death, which contributes to the robustness of ETI. Furthermore, the dual functionality of MPK3/MPK6 cascade in promoting defense and inhibiting photosynthesis potentially allow it to orchestrate the trade-off between plant growth and defense in plant immunity.

## Author summary

Plants follow different strategies to defend themselves against pathogens and activate their immune systems once the pathogens have been detected. One of the responses observed is the inhibition of photosynthesis and the global down-regulation of genes that regulate this process, similar to what is frequently observed in plants under various biotic stress conditions. However, the mechanisms underlying the turning off of the photosynthetic activity and whether this process contributes to plants’ defense against pathogens remain to be determined. In this study, we analyze these mechanisms in *Arabidopsis* plants and show that prolonged activation of MPK3 and MPK6, two kinases critical for pathogen resistance, results in the inhibition of photosynthesis and accumulation of reactive oxygen species (ROS) in the chloroplasts. We find that this response is similar to that observed during pathogen effector-triggered immunity (ETI). Correspondingly, plants that carry mutant versions of MPK3 and MPK6 result in compromised ETI-induced photosynthetic inhibition and chloroplastic ROS accumulation; thus, these two kinases seem to be essential for ETI. Our results suggest that MPK3/MPK6 activation induces a global down-regulation of photosynthesis along with an up-regulation of defense-related genes, and coordinates the growth and defense trade-off in plants.

## Introduction

Plant defense against invading pathogens relies on a two-layered innate immune system. The first is the sensing of pathogen/microbe-associated molecular patterns (PAMPs) by plant pattern recognition receptors (PRRs), which induces a basal level resistance known as PAMP-triggered immunity (PTI) [1–5]. The second line of plant defense is activated by plant resistance (R) protein-mediated detection of pathogenic effectors, also known as effector-triggered immunity (ETI). The major feature of ETI is its robustness against pathogen infection, which is frequently associated with hypersensitive response (HR) cell death [1,3, 6–10].

All identified PRRs are membrane-localized receptor-like protein kinases (RLKs) or receptor-like proteins (RLPs) that detect conserved PAMPs such as bacterial flagellin and lipopolysaccharide or chitin from fungal cell walls [11,12]. Pathogen effectors are sensed by plant R proteins with diverse subcellular locations [13,14]. The majority of plant R proteins are nucleotide-binding site leucine-rich repeat (NBS-LRR) proteins, having a central NBS-ARC domain (ARC: Apaf1, R proteins, and CED-4) and a C-terminal leucine-rich repeat (LRR) domain [14–17]. Based on their N-terminal domains, plant NBS-LRR R proteins (NLRs) are classified into two families, the coiled coil-nucleotide binding site-leucine rich repeat (CNL) family and the Toll/interleukin-1 receptor-nucleotide binding site-leucine rich repeat (TNL) family [14–17]. Unlike PRRs, which detect PAMPs only by direct ligand-receptor recognition, NLRs utilize diverse strategies to detect effectors directly or indirectly. In the direct model, NLRs recognize their cognate effectors by direct protein–protein interactions, while the indirect recognition describes mechanisms by which NLRs sense effectors by monitoring modified self, including the “guard,” the “decoy,” and the “integrated decoy” model [18–20].

Compared to the well-studied effector recognition, the mechanisms underlying the activation of NLRs and their downstream signaling pathways are still poorly understood [14–17]. Current findings suggest that the activity of NLRs undergoes multilayered regulation, including self-inhibition, dimerization or oligomerization, epigenetic and transcriptional regulation, alternative splicing, and proteasome-mediated degradation [14–17]. Despite of the different recognition and activation mechanisms of NLRs and PRRs, ETI and PTI involve a similar set of downstream defense responses, including calcium-mediated signaling, activation of mitogen-activated protein kinases (MAPKs), production of reactive oxygen species (ROS), transcriptional reprogramming, and biosynthesis of antimicrobial compounds [3,4,7,21–28]. However, the responses during ETI have a longer duration and higher magnitude. As a result, ETI was proposed to be an amplified PTI [7]. Recently, it was proposed that plasma membrane–localized CNLs such as Resistance to *Pseudomonas syringae* pv *maculicola* 1 (RPM1), Resistance to *Pseudomonas syringae* 2 (RPS2), and Resistance to *Pseudomonas syringae* 5 (RPS5) trigger downstream defense responses similar to that activated by PRRs during PTI, with the exception of different magnitude and duration [12]. In contrast, ETI mediated by nucleus-localized TNLs, including Resistance to *Pseudomonas syringae* 4/Resistance to *Ralstonia solanacearum* 1 (RPS4/RRS1) and Resistance to *Pseudomonas syringae* 6 (RPS6), which is dependent on Enhanced Disease Susceptibility 1 (EDS1), seems to be more associated with transcriptional reprogramming [12]. This notion was supported by the findings that both plasma membrane–localized PRRs and CNL-type RPS2 can activate MPK3/MPK6 [24]. However, whether TNLs can activate MAPK signaling remains to be determined. Interestingly, EDS1 also contributes to RPS2-conditioned resistance when salicylic acid (SA) biosynthesis is blocked [29], indicating a complicated cross talk between CNL- and TNL-mediated resistance.

Recently, two new immune responses were identified, cell cycle repression and chloroplast stromule formation [30,31]. During ETI, the canonical function of cyclin-dependent kinase inhibitor (CKI)-retinoblastoma (RB)-E2F transcription factor in cell cycle progression is repressed and shifted to promote programmed cell death and transcriptional reprogramming [30]. Chloroplast stromules, dynamic tubular projections of chloroplasts, are strongly induced during plant immunity [31]. Some of the stromules were observed to connect chloroplasts with the nucleus, which was proposed to transport pro-defense signals, e.g., chloroplast-generated ROS, into the nucleus to promote transcriptional reprogramming [31]. At present, the mechanism underlying the generation of ROS in chloroplasts is still unclear. We have previously shown that prolonged activation of SA-induced protein kinase (SIPK) and wound-induced protein kinase (WIPK), the orthologs of *Arabidopsis* MPK6 and MPK3 in tobacco, respectively, inhibits photosynthesis and induces the accumulation of ROS in chloroplasts, which accelerates HR-like cell death in plants under light [32]. Later, chloroplast-originated ROS was implicated in promoting localized cell death in tobacco during nonhost interaction [33]. Because HR cell death can be uncoupled from ETI [34], i.e., host cell death is not the cause for resistance [7], it remains to be determined whether MAPK signaling and chloroplast-originated ROS accumulation contribute to ETI.

In *Arabidopsis*, MPK3, MPK6, MPK4, and MPK11 are rapidly activated during PTI and ETI [24,28,35]. They play critical roles in multiple plant defense responses, including activation of defense gene expression, induction of phytoalexin biosynthesis, and stomatal immunity [28,36–40]. Light is known to be critical to plant defense [41–43]. It has been known for decades that photosynthetic inhibition, including inhibition of photosystem II (PSII) activity, reduction of CO_2_ fixation, and global down-regulation of photosynthetic genes, occurs after pathogen infection [44–50]. However, it is unclear whether the photosynthetic inhibition is a passive response due to pathogen infection or an active response regulated by host signaling pathways, i.e., whether photosynthetic inhibition is a part of plant immunity and contributes to plant disease resistance.

In this study, we demonstrate that both CNL and TNL NLR-mediated ETI induce prolonged activation of MPK3/MPK6, which contributes to the rapid and global inhibition of photosynthesis at multiple levels and the generation of ROS in chloroplasts. Loss-of-function of MPK3/MPK6 signaling compromises effector-triggered inhibition of photosynthetic activities, accumulation of ROS in chloroplasts, HR cell death, and pathogen resistance. Furthermore, it was discovered that inhibition of photosynthetic activities and chloroplastic ROS accumulation can mutually enhance each other. Based on these findings, we conclude that MPK3/MPK6-mediated active photosynthetic inhibition is a part of *Arabidopsis* immune response and plays a positive role during ETI.

## Results

### MPK3/MPK6 activation induces global down-regulation of genes related to photosynthesis

**Fig 1:**
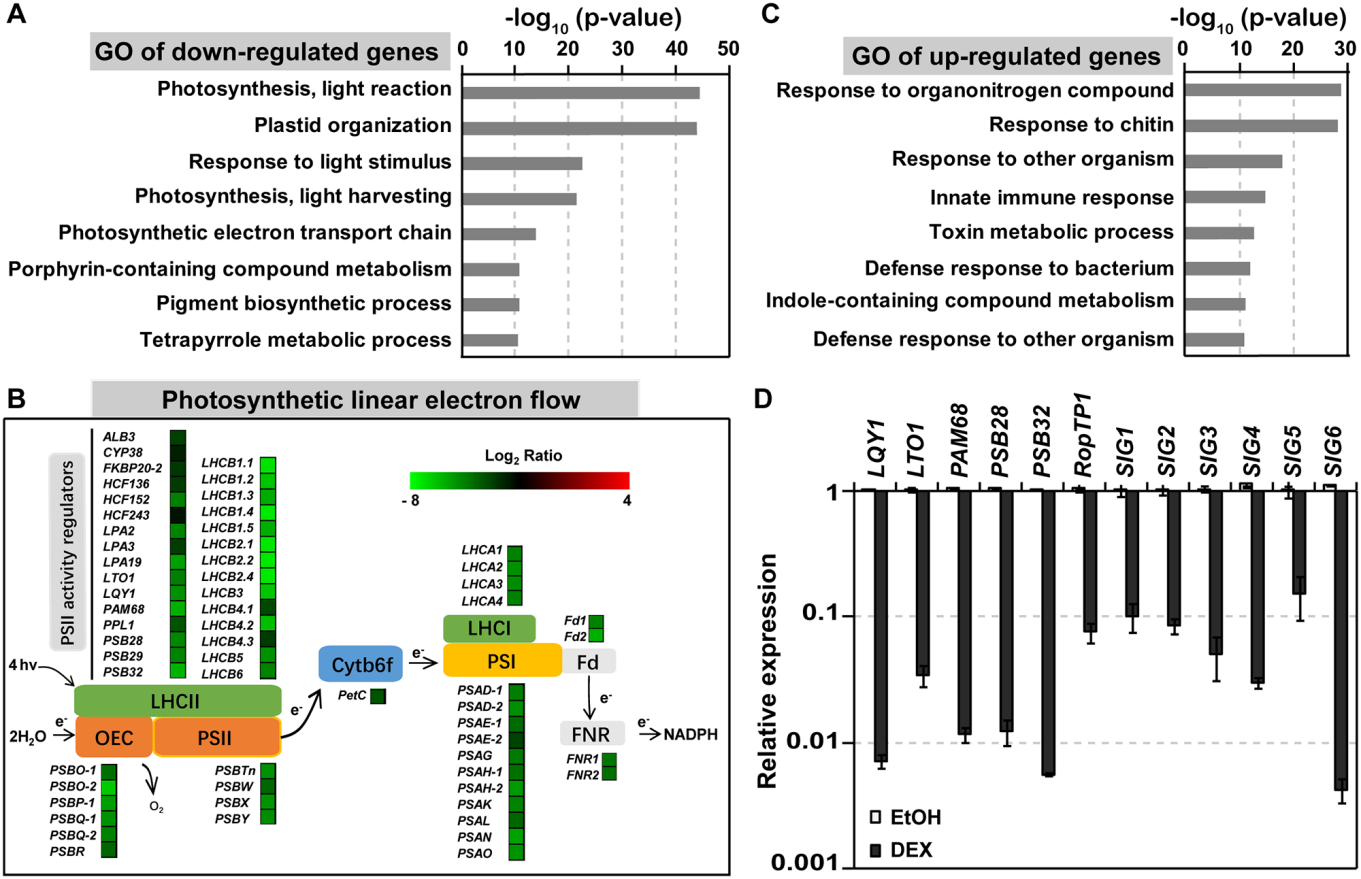
Activation of MPK3/MPK6 globally down-regulates photosynthetic genes. (**A**) GO enrichment of 2,977 down-regulated genes (log_2_ ratio ≤ −3) after MPK3/MPK6 activation. RNA-seq analysis was performed using 12-d-old *DD* seedlings treated with 5 μM DEX for 0 and 6 h under light. Transcript levels were fold changes relative to the 0 h sample. GO analysis was carried out by using DAVID online tool [57,58]. Enrichment scores are shown as −log_10_ (*p*-value). (**B**) Schematic diagram of photosynthetic linear electron flow. Expression levels of genes involved in photosystem assembly and repair are shown as a heat map. (**C**) GO enrichment of 1,039 up-regulated genes (log_2_ ratio ≤ −3) after MPK3/MPK6 activation. Enrichment scores are shown as −log_10_ (*p*-value). (**D**) Activation of MPK3/MPK6 induces drastic down-regulation of photosynthesis-related genes. Twelve-d-old *DD* plants grown in liquid medium were treated with EtOH or 5 μM DEX for 8 h under light. Transcript levels were quantified by real-time PCR and presented as fold changes relative to 0 h samples. Values are means ± SD, *n* = 3. *EF1a* was used as internal control, *n* = 3. The numerical values used to construct panels A–D can be found in S1 Data. See also S1 Table. Cytb6f, cytochrome b6f; DAVID, Database for Annotation, Visualization and Integrated Discovery; *DD*, *GVG-NtMEK2*^*DD*^; DEX, dexamethasone; *EF1a*, elongation factor *1a*; EtOH, ethanol; Fd, ferredoxin; FNR, ferredoxin-NADP^+^ reductase; GO, gene ontology; LHCI, light-harvesting complex I; LHCII, light-harvesting complex II; MPK, mitogen-activated protein kinase; OEC, oxygen-evolving complex; PSI, photosystem I; PSII, photosystem II; RNA-seq, RNA sequencing.

Inhibition of photosynthesis occurs in plants under a variety of abiotic and biotic stresses [44,51]. At present, it is unclear whether the inhibition is a passive response caused by stresses/pathogens or a response actively regulated by host signaling pathways, and if so, what the outcomes/functions are of such active inhibition. In our previous study, we found that activation of SIPK and WIPK, two stress-responsive MPKs in tobacco, causes rapid and strong inhibition of CO_2_ fixation [32]. To elucidate the underlying mechanism, we utilized the *Arabidopsis* system and profiled the gene expression in the conditional gain-of-function *Arabidopsis GVG-NtMEK2*^*DD*^ (abbreviated as *DD*) transgenic plants. In this system, dexamethasone (DEX) treatment induces the expression of NtMEK2^DD^, a constitutively active variant of *Nicotiana tabacum* MAP kinase kinase 2 (NtMEK2), which in turn activates specifically the endogenous MPK3 and MPK6 in *Arabidopsis* [52,53]. RNA sequencing (RNA-seq) profiling revealed a total of 2,984 genes that were down-regulated (log_2_ < −3) and 1,042 genes up-regulated (log_2_ > 3) at 6 h after DEX treatment (S1 Table). Genes involved in photosynthesis, including photosynthetic light harvesting, light reaction, electron transport, and dark reaction, were highly enriched in the down-regulated genes (Fig 1A and 1B). The up-regulated genes were mainly enriched in genes involved in plant–pathogen and plant–environment interactions and secondary metabolism (Fig 1C). Up-regulation of defense genes, including those involved in phytoalexin biosynthesis, is consistent with our previous reports [28,53–56]. Down-regulation of selected photosynthetic genes was further confirmed by quantitative reverse transcription-polymerase chain reaction (RT-PCR). As shown in Fig 1D, expression of genes involved in PSII repair (*LQY1* and *LTO1*), PSII assembly (*PAM68*), PSII stabilization (*PSB32*), and transcription in chloroplasts (e.g., *SIG1*–*SIG6*) were all decreased drastically after MPK3/MPK6 activation.

### MPK3/MPK6 activation causes photosynthetic inhibition and ROS accumulation in chloroplasts

**Fig 2:**
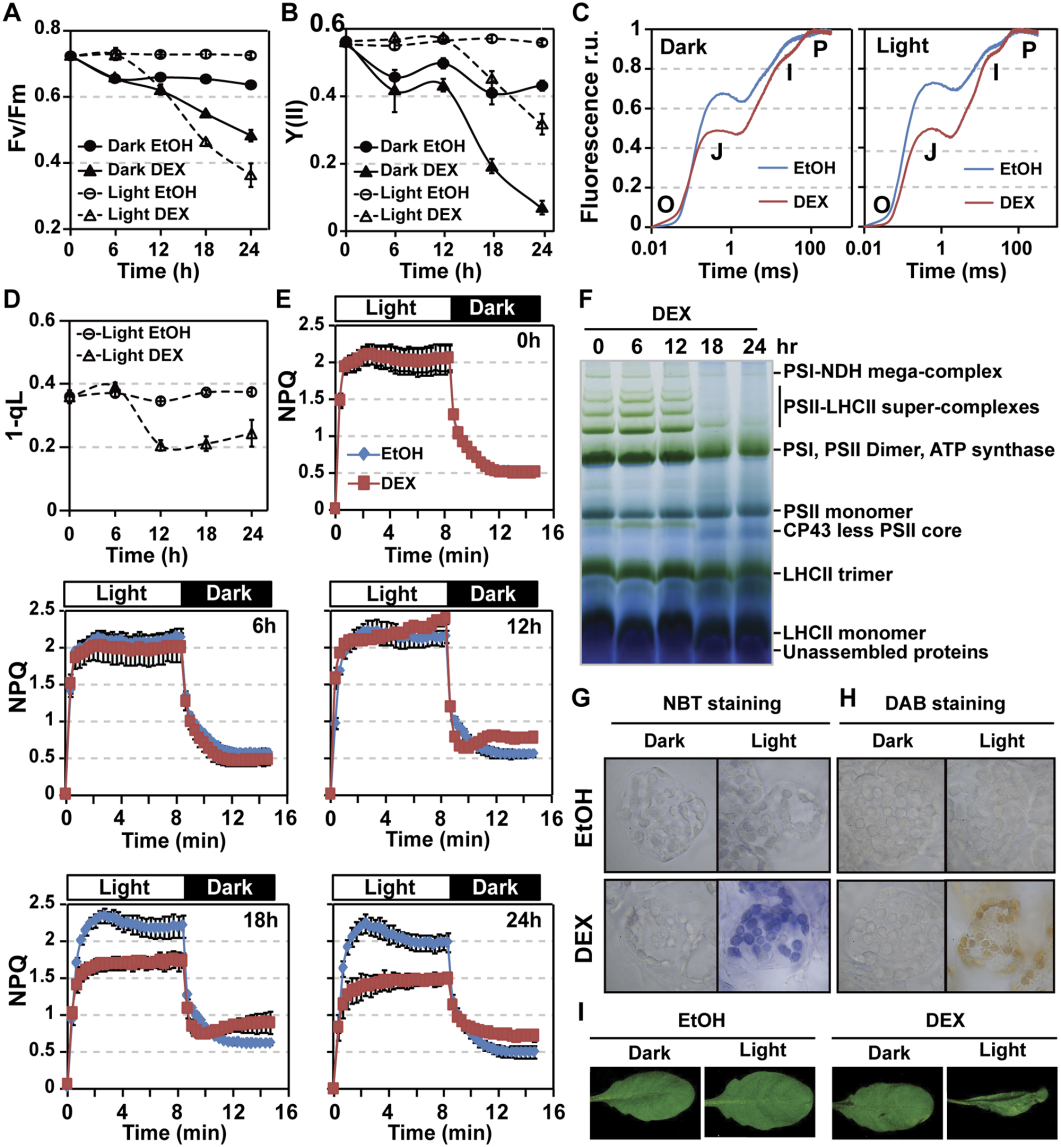
Activation of MPK3/MPK6 induces multilayered photosynthetic inhibition ROS accumulation in chloroplasts. (**A** and **B**) Activation of MPK3/MPK6 induces PSII inhibition. Fv/Fm (A) and Y(II) (B) were measured using soil-grown *DD* plants spray treated with EtOH (solvent control) or 15 μM DEX for indicated periods of time. Values are means ± SD, *n* = 8. (**C**) Changes of O-J-I-P curve induced by MPK3/MPK6 activation. Normalized fluorescence units were shown. Soil-grown *DD* plants were spray treated with EtOH or 15 μM DEX and kept in dark or light for 12 h. Representative measurements for each treatment were shown. (**D**) MPK3/MPK6 activation affects PQ pool redox status. Values are means ± SD, *n* = 8. (**E**) Activation of MPK3/MPK6 causes decreased NPQ at late stage. After treatment with EtOH or 15 μM DEX for indicated times under light, NPQ induction was carried out by using a light intensity of 610 μmol m^−2^ s^−1^ for 7 min and followed by an 8-min relaxation period in dark. Values are means ± SD, *n* = 6. (**F**) Activation of MPK3/MPK6 induces a decrease in PSII-LHCII super-complexes and an increase in CP43-less PSII core complex. Twelve-d-old *DD* plants grown in liquid medium were treated with 5 μM DEX for indicated times. Thylakoid membranes were isolated and solubilized with 1% dodecyl maltoside. Samples equivalent to 8 μg of chlorophyll were loaded to a BN-PAGE. (**G** and **H**) Activation of MPK3/MPK6 induces light-dependent accumulation of ROS in chloroplasts. *DD* plants grown in liquid medium were treated with EtOH solvent control or 5 μM DEX and kept in dark or light for 8 h. Cellular ${\text{O}}_{2}^{•-}$(G) and H_2_O_2_ (H) were visualized by NBT and DAB staining, respectively. (**I**) Light accelerates MPK3/MPK6 activation-induced HR-like cell death. Soil-grown *DD* plants were first spray treated with EtOH solvent control or 15 μM DEX and then kept in dark or under light for 36 h. The numerical values used to construct panels A–E can be found in S1 Data. See also S1 and S2 Figs. BN-PAGE, blue native polyacrylamide gel electrophoresis; CP43, photosystem II chlorophyll protein of 43 kDa; DAB, 3,3′-diaminobenzidine; *DD*, *GVG-NtMEK2*^*DD*^; DEX, dexamethasone; EtOH, ethanol; HR, hypersensitive response; LHCII, light-harvesting complex II; MPK, mitogen-activated protein kinase; NBT, nitroblue tetrazolium; NDH, NADH dehydrogenase-like; NPQ, non-photochemical quenching; PQ, plastoquinol; PSI, photosystem I; PSII, photosystem II; ROS, reactive oxygen species; r.u., relative unit.

To determine the physiological consequences of the inhibition of photosynthetic genes, we measured PSII activity using chlorophyll fluorescence techniques [59]. As shown in Fig 2A and 2B, the maximal PSII activity parameter Fv/Fm and effective PSII operating efficiency parameter Y(II) both decreased upon MPK3/MPK6 activation. We next measured the fast chlorophyll fluorescence kinetics, also known as O-J-I-P curve [60]. The J-I rise was lower in DEX-treated *DD* plants than in ethanol solvent control (Fig 2C), indicating a reduced plastoquinol (PQ) reduction after MPK3/MPK6 activation. We next measured PQ redox status parameter 1-qL. Consistent with a decrease in PSII activity, a more oxidized PQ pool, reflected by the decrease of 1-qL, was detected (Fig 2D).

NPQ dissipates light energy as heat to protect PSII from photodamage [61,62]. We found that NPQ induction by high light (610 μmol m^−2^ s^−1^) was not affected at the early stage of MPK3/MPK6 activation but decreased significantly at 18 h and 24 h after DEX treatment (Fig 2E). The decreased NPQ at 18 h and 24 h is likely to be a consequence of PSII inhibition rather than an active down-regulation of NPQ. The decreased NPQ may accelerate PSII inhibition due to impaired dissipation of light energy.

We next examined PSII inhibition using blue native polyacrylamide gel electrophoresis (BN-PAGE), by which changes of thylakoid membrane photosynthetic complexes can be visualized. There was a decrease in PSII-LHCII super-complexes and an increase in CP43-less PSII core complex in both BN-PAGE (Fig 2F) and two-dimensional sodium dodecyl sulfate (SDS)-PAGE analyses (S1 Fig), which correlates well with PSII inhibition. Collectively, the changes in photosynthetic parameters and complexes were found to be associated with the down-regulation in mRNA levels of photosynthetic genes after MPK3/MPK6 activation in *Arabidopsis* (Fig 1A). Together with our previous report that SIPK/WIPK activation inhibits photosynthetic activities in tobacco [32], we can conclude that inhibition of photosynthesis after the activation of pathogen-responsive MPK3/MPK6 in *Arabidopsis* or their orthologs in other plant species is a common response in plants.

Photosynthesis inhibition in plants frequently leads to the accumulation of ROS [63,64]. As shown in Fig 2G and S2 Fig, nitroblue tetrazolium (NBT) staining revealed an accumulation of superoxide (${\text{O}}_{2}^{•-}$) in the chloroplasts of *DD* plants after MPK3/MPK6 activation. This only occurred in plants kept under light. In the dark, no ${\text{O}}_{2}^{•-}$ accumulation was observed. In chloroplasts, superoxide can be quickly converted to H_2_O_2_ under the action of superoxide dismutase. Using 3,3′-diaminobenzidine (DAB) staining, we indeed detected the accumulation of H_2_O_2_ in chloroplasts after MPK3/MPK6 activation in a light-dependent manner (Fig 2H and S2 Fig). Consistent with our previous report using tobacco, MPK3/MPK6 activation-induced HR-like cell death in *Arabidopsis* was also light dependent (Fig 2I). It is worth noting that MPK3/MPK6 activation–induced PSII inhibition is independent of light, although it is delayed in the absence of light (Fig 2A and 2C), suggesting that ROS accumulation in chloroplasts may accelerate PSII inhibition and HR-like cell death in *DD* plants.

### Long-lasting MPK3/MPK6 activation is needed to induce photosynthetic inhibition

**Fig 3:**
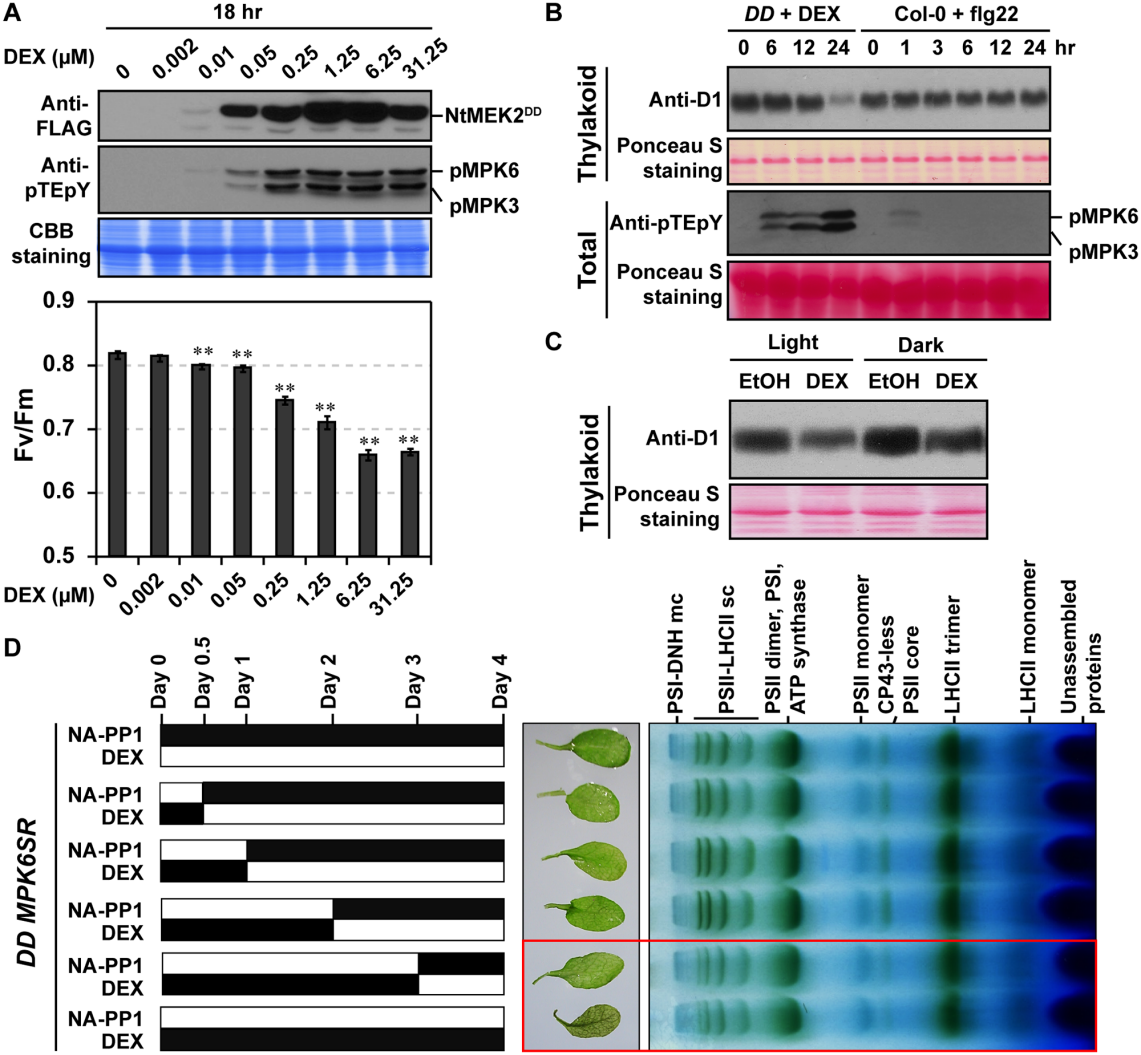
The amplitude and duration of MAPK activation regulate photosynthetic inhibition. (**A**) Controlling the amplitude of MPK3/MPK6 activation in *DD* plants by spraying different concentrations of DEX. Soil-grown *DD* plants were spray treated with different concentrations of DEX. At 9 h after DEX spray, NtMEK2^DD^ expression and MPK3/MPK6 activation were detected by immunoblot analysis using anti-FLAG and anti-pTEpY, respectively. Fv/Fm was measured at 18 h after DEX treatment. Values are means ± SD, *n* = 6, **P ≤ 0.001. The numerical values used to construct panel A can be found in S1 Data. (**B**) Transient activation of MPK3/MPK6 after flg22 treatment does not induce a decrease in PSII core D1 protein. Twelve-d-old *DD* and Col-0 plants grown in liquid medium were treated with 5 μM DEX and 200 nM flg22 for indicated times, respectively. Thylakoid membranes were isolated and solubilized with 1% dodecyl maltoside. Samples equivalent to 2 μg of chlorophyll were loaded to a 12% SDS-PAGE with 6 M urea. Anti-D1 was used to detect D1 abundance. MAPK activation was detected by immunoblot analysis using anti-pTEpY antibody. Ponceau S staining was used to show equal loading. (**C**) Twelve-d-old *DD* and Col-0 plants grown in ½ MS agar plates were treated with 5 μM DEX or EtOH (solvent control) and then kept in dark or under light for 12 h. D1 protein abundance in thylakoid membrane preparations was detected by immunoblot analysis using anti-D1 antibody. Ponceau S staining was used to show equal loading. (**D**) Prolonged, but not transient, MAPK activation induces photosynthetic inhibition. Twelve-d-old *DD MPK6SR* plants grown in liquid medium were first treated with 5 μM DEX. Seedlings were washed three times with ½ MS medium to remove DEX before the addition of 10 μM NA-PP1. Black bars indicate the durations of DEX or NA-PP1 treatment. Thylakoid membranes were isolated and solubilized with 1% dodecyl maltoside. Samples equivalent to 8 μg of chlorophyll were loaded to a BN-PAGE. See also S3 Fig. BN-PAGE, blue native polyacrylamide gel electrophoresis; CBB, Coomassie brilliant blue; Col-0, Columbia-0; CP43, photosystem II chlorophyll protein at 43 kDa; *DD*, *GVG-NtMEK2*^*DD*^; DEX, dexamethasone; DNH, NADH dehydrogenase-like; EtOH, ethanol; FLAG, an octapeptide; flg22, a 22 amino acids flagellin fragment; LHCII, light-harvesting complex II; mc, mega-complex; MPK, mitogen-activated protein kinase; MS, Murashige and Skoog medium; NA-PP1, 4-amino-1-tert-butyl-3-(1’-naphthyl)pyrazolo[3,4-d]pyrimidine; pMPK, phosphorylated MPK; PSI, photosystem I; PSII, photosystem II; pTEpY, dually phosphorylated Thr/Glu/Tyr peptide; sc, super-complex; SDS-PAGE, sodium dodecyl sulfate-PAGE.

Previous studies showed that MPK3/MPK6 activation is transient during PTI and is prolonged during CNL-type RPS2-mediated ETI [24]. To examine the amplitude of MPK3/MPK6 activation in regulating photosynthetic inhibition, *DD* plants were treated with increasing concentrations of DEX. As shown in Fig 3A, there was a correlation between the level of PSII inhibition and the amplitude of MPK3/MPK6 activation. Prolonged MPK3/MPK6 activation in *DD* caused a drastic photosynthetic inhibition, as demonstrated by western blot detection of PSII core proteins D1 (Fig 3B), while no photosynthetic inhibition was observed in Columbia-0 (Col-0) plants treated with a 22 amino acids flagellin fragment (flg22), which induced a transient MPK3/MPK6 activation (Fig 3B and S3 Fig). Moreover, MPK3/MPK6 activation-induced decrease of D1 protein is also independent of light (Fig 3C), which correlates well with the measured chlorophyll fluorescence in dark (Fig 2A–2C). To further test the duration of MAPK activation in regulating photosynthetic inhibition, we crossed *DD* transgene into *MPK6SR*, a chemical-genetically rescued *mpk3 mpk6* double mutant system [38,65], to generate *DD MPK6SR* (genotype: *GVG-NtMEK2*^*DD*^ *mpk3 mpk6 P*_*MPK6*_:*MPK6*^*YG*^). In *DD MPK6SR* plants, MPK6 can be activated by DEX and inhibited by 4-amino-1-tert-butyl-3-(1’-naphthyl)pyrazolo[3,4-d]pyrimidine (NA-PP1), a specific inhibitor of the sensitized *MPK6*^*YG*^. As revealed by BN-PAGE analysis, short-term MPK6 activation failed to induce photosynthetic inhibition (Fig 3D), demonstrating that prolonged, but not transient, MAPK activation is required for photosynthetic inhibition. It was noted that photosynthetic inhibition in *DD MPK6SR* plants was slower in comparison to *DD* plants after DEX treatment, which may be due to the lack of *MPK3*, or reduced expression of NtMEK2^DD^ as a result of gene silencing, or both. Nonetheless, it clearly demonstrates that long-lasting, but not transient, MPK3/MPK6 activation induces photosynthetic inhibition.

### ETI, but not PTI, induces prolonged MAPK activation, photosynthetic inhibition, and ROS accumulation in chloroplasts

To determine the involvement of MPK3/MPK6 in photosynthetic inhibition during plant immunity, we first measured the kinetics of photosynthetic parameters in wild-type plants infiltrated with *Pseudomonas syringae* pv. *tomato DC3000* carrying empty vector (*Pst-EV*), *Pst-AvrRpt2*, a *Pst* strain carrying the avirulence effector recognized by RPS2 (*AvrRpt2*) effector gene, or *Pst-hrcC*^−^, a *Pst* strain carrying a mutation in *hrcC* gene. *Pst-AvrRpt2* triggers both PTI and CNL-type *RPS2*-dependent ETI in *Arabidopsis*. Due to the lack of functional type-III secretion system, *Pst-hrcC*^−^ cannot deliver effectors into plant cells and only induces PTI.

**Fig 4:**
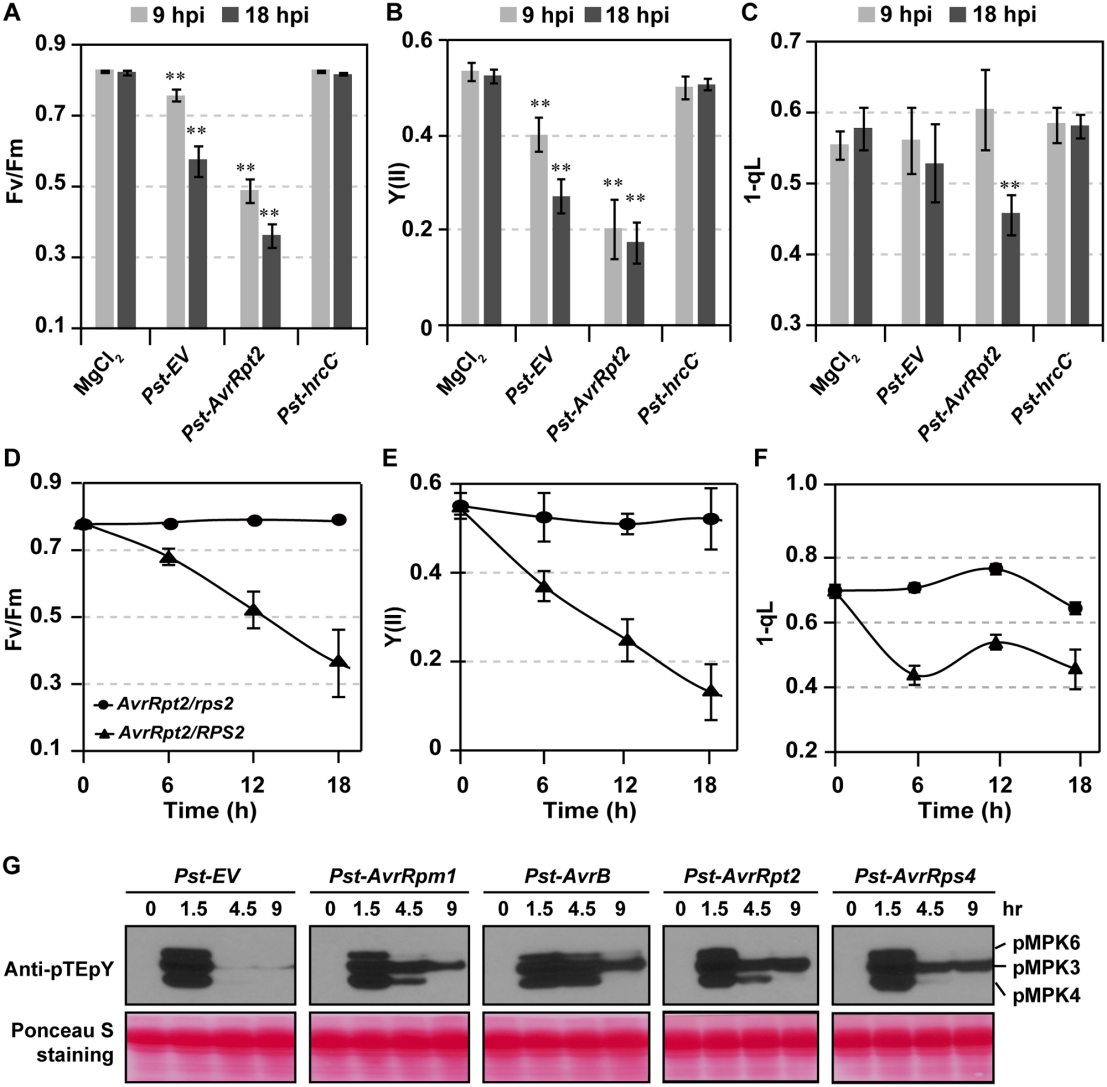
ETI induces strong PSII inhibition and prolonged MAPK activation. (**A**–**C**) Photosynthetic parameters are differentially affected by different *Pst* strains. Four-wk-old Col-0 plants were infiltrated with 10 mM MgCl_2_ (mock), *Pst-EV*, *Pst-AvrRpt2*, or *Pst-hrcC*^−^ (OD_600_ = 0.2) and then were kept under light with a transparent lid. Photosynthetic parameters, including Fv/Fm (A), Y(II) (B), and 1-qL (C), were measured at 9 and 18 hpi. Values are means ± SD, *n* = 5–8, **P ≤ 0.001. (**D**–**F**) AvrRpt2-triggered immunity induces drastic PSII inhibition. Four-wk-old soil-grown *GVG-AvrRpt2* transgenic plants in Col-0 (*AvrRpt2/RPS2*) or *rps2* mutant background (*AvrRpt2/rps2*) were spray treated with 15 μM DEX. Photosynthetic parameters, including Fv/Fm (D), Y(II) (E), and 1-qL (F), were measured at indicated times. Values are means ± SD, *n* = 4–8. The numerical values used to construct panels A–F can be found in S1 Data. (**G**) ETI mediated by both CNL- and TNL-type NLRs induces prolonged MAPK activation. Four-wk-old Col-0 plants were infiltrated with *Pst* carrying *EV*, *AvrRpm1*, *AvrB*, AvrRpt2, or *AvrRps4* (OD_600_ = 0.02) for indicated time points. MPK3/MPK6 activation was detected by anti-pTEpY antibody. See also S4 and S5 Figs. *AvrB*, avirulence protein B; *AvrRpm1*, avirulence effector recognized by RPM1; *AvrRpt2*, avirulence effector recognized by RPS2; *AvrRps4*, avirulence effector recognized by RPS4; CNL, coiled coil-nucleotide binding site-leucine rich repeat; Col-0, Columbia-0; DEX, dexamethasone; ETI, effector-triggered immunity; EV, empty vector; *GVG-AvrRpt2*, DEX-inducible promoter-driven *AvrRpt2*; hpi, hours post inoculation; *hrcC*^*−*^, outer membrane type III secretion protein HrcC mutant; MPK, mitogen-activated protein kinase; NLR, nucleotide-binding leucine-rich repeat; OD, optical density; pMPK, phosphorylated MPK; PSII, photosystem II; *Pst*, *Pseudomonas syringae* pv *tomato*; pTEpY, dually phosphorylated Thr/Glu/Tyr peptide; *RPS2*, Resistance to *Pseudomonas syringae* 2; TNL, Toll/interleukin-1 receptor-nucleotide binding site-leucine rich repeat.

Similar to the gain-of-function activation of MPK3/MPK6 in *DD* plants, *Pst-AvrRpt2* induced drastic reductions in Fv/Fm (Fig 4A and S4 Fig), Y(II) (Fig 4B and S4 Fig), and 1-qL (Fig 4C). The decreases in Fv/Fm and Y(II) after *Pst-EV* inoculation were much slower and delayed in comparison to after *Pst-AvrRpt2* inoculation (Fig 4A and 4B and S4 Fig). Interestingly, *Pst-hrcC*^−^, which only induces PTI, had no effect on any measured chlorophyll fluorescence parameters (Fig 4A–4C and S4 Fig), indicating that PTI is not sufficient to induce photosynthetic inhibition. Consistent with this conclusion, flg22 infiltration failed to induce change in photosynthetic parameters and decrease of D1 protein (Fig 3B and S4 Fig). In addition, no photosynthetic changes were detected after infiltration of a nonpathogenic strain, *P*. *fluorescens*, carrying empty vector [66], while *P*. *fluorescens* carrying *AvrRpm1*, which triggers CNL-type RPM1-mediated ETI, induced strong photosynthetic inhibition (S4 Fig). These results further support that ETI, but not PTI, induces photosynthetic inhibition.

The interaction between *Pst-AvrRpt2* and *Arabidopsis* is complex, involving both virulent and avirulent effectors that can induce effector-triggered susceptibility (ETS) and ETI, respectively, besides PTI. To determine whether ETI is sufficient to induce PSII inhibition, we utilized the DEX-inducible promoter-driven *AvrRpt2* (*GVG-AvrRpt2*) transgenic plants [67]. DEX treatment of *GVG-AvrRpt2* plants is sufficient to activate MPK3/MPK6 in a *RPS2*-dependent manner [25]. Similar to MPK3/MPK6 activation and *Pst-AvrRpt2* inoculation, DEX treatment of *GVG-AvrRpt2* plants was sufficient to induce drastic PSII inhibition (Fig 4D and 4E) and PQ pool oxidation (Fig 4F). Similarly (Fig 2G and 2H), induction of *AvrRpt2* expression also led to the accumulation of ${\text{O}}_{2}^{•-}$ and H_2_O_2_ in chloroplasts (S5 Fig). In *rps2* mutant background, AvrRpt2-induced photosynthetic inhibition and accumulation of ROS in chloroplasts were abolished (Fig 4D–4F and S5 Fig), demonstrating that AvrRpt2 effector is sufficient to induce photosynthetic inhibition and ROS generation in an RPS2-dependent fashion.

We also tested MAPK activation and chlorophyll fluorescence parameters after infiltrating with *Pst-AvrRpm1*, *Pst-AvrB* and *Pst-AvrRps4*. Among the four tested effectors, AvrRpt2 and AvrRpm1 induce CNL NLR-dependent ETI [68–72], AvrB induces both CNL and TNL NLR-dependent ETI [70,72,73], and AvrRps4 induces TNL NLR-dependent ETI [74,75]. Interestingly, we found TNL ETI also induces prolonged MAPK activation and PSII inhibition (Fig 4G and S6 Fig), indicating that MAPK signaling and its activation-mediated photosynthetic inhibition and chloroplastic ROS accumulation are essential for both CNL and TNL NLR-conditioned ETI.

### MPK3 and MPK6 are required for both CNL- and TNL-mediated ETI

**Fig 5:**
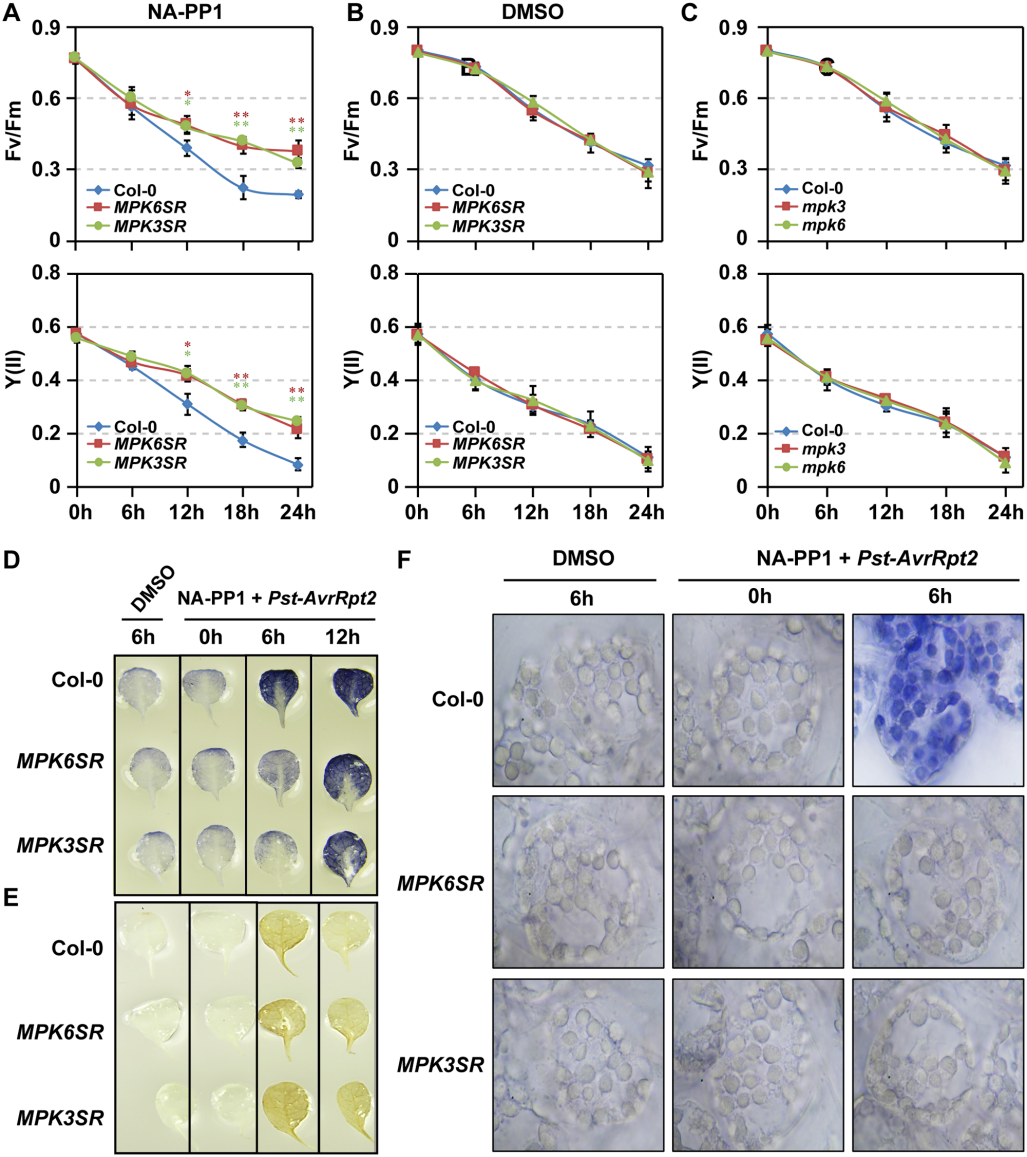
MPK3 and MPK6 are required for ETI-induced PSII inhibition and ROS accumulation in chloroplasts. (**A** and **B**) *Pst-AvrRpt2*–induced PSII inactivation is partially compromised in *mpk3 mpk6* double mutant. Four-wk-old soil-grown Col-0, *MPK6SR*, and *MPK3SR* plants were first spray treated with NA-PP1 (10 μM) or DMSO solvent control for 2 h and then infiltrated with *Pst-AvrRpt2* (OD = 0.2). Values are means ± SD, *n* = 4, 0.01 ≤ *P ≤ 0.001 and **P ≤ 0.001. (**C**) Normal *Pst-AvrRpt2*–induced PSII inactivation in *mpk3* and *mpk6* single mutants. Four-wk-old soil-grown Col-0, *mpk3*, and *mpk6* plants were directly infiltrated with *Pst-AvrRpt2* (OD = 0.2). Fv/Fm and Y(II) were measured at indicated time points. Values are means ± SD, *n* = 4. The numerical values used to construct panels A–C can be found in S1 Data. (**D**–**F**) MPK3 and MPK6 are required for *Pst-AvrRpt2*–induced ${\text{O}}_{2}^{•-}$, but not H_2_O_2_, accumulation. Twelve-d-old Col-0, *MPK6SR*, and *MPK3SR* plants grown in liquid medium were first treated with 2 μM NA-PP1 or DMSO (mock) for 1 h. They were then treated with *Pst-AvrRpt2* (OD_600_ = 0.02) for indicated periods of time. ${\text{O}}_{2}^{•-}$ and H_2_O_2_ accumulation were detected by NBT and DAB staining, respectively. *AvrRpt2*, avirulence effector recognized by RPS2; Col-0, Columbia-0; DAB, 3,3′-diaminobenzidine; ETI, effector-triggered immunity; MPK, mitogen-activated protein kinase; NA-PP1, 4-amino-1-tert-butyl-3-(1’-naphthyl)pyrazolo[3,4-d]pyrimidine; NBT, nitroblue tetrazolium; OD, optical density; ${\text{O}}_{2}^{•-}$, superoxide; *Pst*, *Pseudomonas syringae* pv *tomato*; PSII, photosystem II; ROS, reactive oxygen species.

To determine whether MPK3/MPK6 are required for ETI-induced photosynthetic inhibition and ROS accumulation in chloroplasts, we utilized the newly generated chemical-genetically rescued *mpk3 mpk6* double mutant systems [38,65]. Both *MPK6SR* (genotype: *mpk3 mpk6 P*_*MPK6*_:*MPK6*^*YG*^) and *MPK3SR* (genotype: *mpk3 mpk6 P*_*MPK3*_:*MPK3*^*TG*^) were tested. As is shown in Fig 5A, *Pst-AvrRpt2*–induced PSII inhibition was partially impaired in *MPK6SR* and *MPK3SR* plants after pretreatment with NA-PP1, a specific inhibitor of the sensitized *MPK6*^*YG*^ and *MPK3*^*TG*^, demonstrating that MPK3 and MPK6 are required for the fast and drastic PSII inhibition triggered by ETI activation. No differences were observed in *MPK6SR* and *MPK3SR* plants pretreated with DMSO (Fig 5B), or *mpk3* and *mpk6* single mutants (Fig 5C), demonstrating that MPK3 and MPK6 function redundantly in mediating ETI-induced PSII inhibition.

We next examined *MPK6SR* and *MPK3SR* plants inoculated with *Pst-AvrRpt2* to determine whether MPK3 and MPK6 are required for the ETI-mediated increase in ${\text{O}}_{2}^{•-}$/H_2_O_2_. As shown in Fig 5D and 5F, *Pst-AvrRpt2*–induced ${\text{O}}_{2}^{•-}$ accumulation in chloroplasts was delayed in NA-PP1–treated *MPK6SR* and *MPK3SR*, which correlates well with the reduced PSII inhibition in NA-PP1–treated *MPK6SR* and *MPK3SR* plants (Fig 5A). These results suggest that MPK3/MPK6 are involved in ETI-induced ROS accumulation in chloroplasts. However, although MPK3/MPK6 activation can induce both accumulation of H_2_O_2_ and ${\text{O}}_{2}^{•-}$ (Fig 2G and 2H and S2 Fig), *Pst-AvrRpt2*–induced H_2_O_2_ accumulation was not affected in *MPK6SR* and *MPK3SR* (Fig 5E), possibly due to the complicated enzymatic and nonenzymatic conversion of ${\text{O}}_{2}^{•-}$ to H_2_O_2_ and/or H_2_O_2_ decomposition.

**Fig 6:**
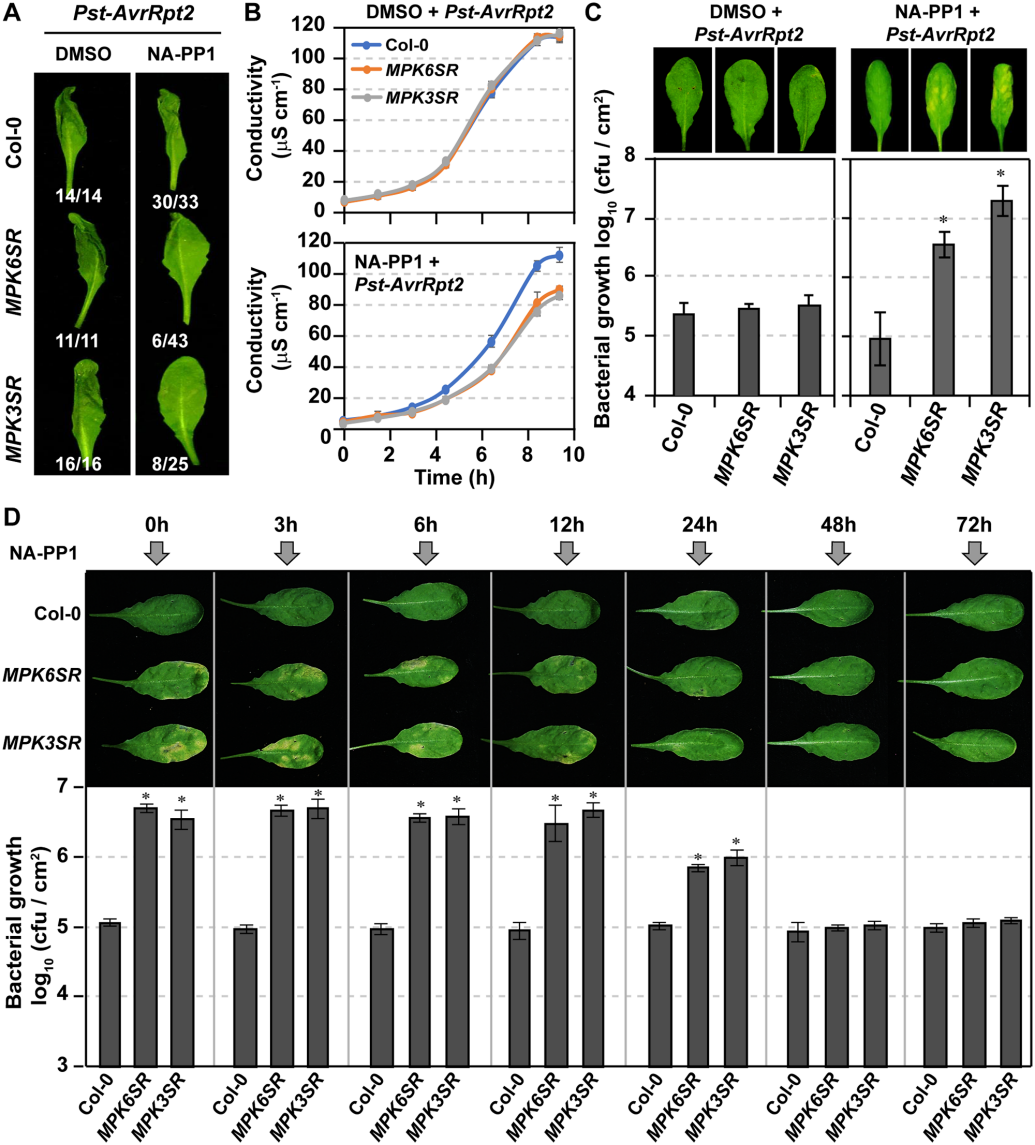
Prolonged MPK3/MPK6 activation is essential to ETI. (**A**) MPK3 and MPK6 are required for *Pst-AvrRpt2*–induced HR cell death. Soil-grown Col-0, *MPK6SR*, and *MPK3SR* plants were first spray treated with 10 μM NA-PP1 for 2 h and then infiltrated with *Pst-AvrRpt2* (OD = 0.02). Photos were taken at 18 hpi. Numbers are ratios of leaves with HR phenotype. (**B**) MPK3 and MPK6 are required for *Pst-AvrRpt2*–induced ion leakage. Soil-grown Col-0, *MPK6SR*, and *MPK3SR* plants were first spray treated with 10 μM NA-PP1 for 2 h; leaf discs were punched and then infiltrated with *Pst-AvrRpt2* (OD = 0.02) by vacuum. Leaf discs were then transferred to GC vials containing 2 μM NA-PP1 or DMSO. Ion leakage was measured as increase in conductivity. Values are means ± SD, *n* = 4. (**C**) MPK3 and MPK6 are required for disease resistance against *Pst-AvrRpt2*. Col-0, *MPK6SR*, and *MPK3SR* plants grown in soil were first spray treated with 10 μM NA-PP1 or DMSO and then infiltrated with *Pst-AvrRpt2* (OD_600_ = 0.0005). NA-PP1 or DMSO was sprayed again at 1.5 dpi. *Pst-AvrRpt2* growth was quantified at 3 dpi. Values are means ± SD, *n* = 3, 0.01 ≤ *P ≤ 0.001. (**D**) Prolonged activation of MPK3/MPK6 is required for ETI-mediated resistance. Col-0, *MPK6SR*, and *MPK3SR* plants grown in soil were infiltrated with *Pst-AvrRpt2* (OD_600_ = 0.0005). NA-PP1 was sprayed at indicated time points. *Pst-AvrRpt2* growth was quantified at 3 dpi. Values are means ± SD, *n* = 3, *P ≤ 0.001. The numerical values used to construct panels B–D can be found in S1 Data. See also S6 Fig. *AvrRpt2*, avirulence effector recognized by RPS2; Col-0, Columbia-0; ETI, effector-triggered immunity; GC, gas chromatography; hpi, hours post inoculation; HR, hypersensitive response; MPK, mitogen-activated protein kinase; NA-PP1, 4-amino-1-tert-butyl-3-(1’-naphthyl)pyrazolo[3,4-d]pyrimidine; OD, optical density; *Pst*, *Pseudomonas syringae* pv *tomato*.

Although HR-like cell death after the activation of MPK3/MPK6 or their orthologs in tobacco was detailed more than a decade ago [52,76,77], it is still unknown whether pathogen-induced HR cell death requires this MAPK cascade. As a result, we examined HR cell death in *MPK3SR* and *MPK6SR* during ETI after *Pst-AvrRpt2* inoculation. As shown in Fig 6A and 6B, HR cell death and ion leakage were impaired in NA-PP1–, but not DMSO–, treated *MPK6SR* and *MPK3SR* plants. Associated with this, we also observed compromised resistance to *Pst-AvrRpt2* in NA-PP1–treated *MPK6SR* and *MPK3SR* plants (Fig 6C). Associated with the high titer of *Pst-AvrRpt2* growth in *Arabidopsis*, leaf chlorosis was observed (Fig 6C), consistent with the breach of plant ETI in the loss-of-function *mpk3 mpk6* double mutant system. In solvent DMSO-treated controls, the growth of *Pst-AvrRpt2* was suppressed, demonstrating an effective ETI (Fig 6C). These results demonstrate that MPK3 and MPK6 function redundantly and are required for ETI.

We also measured PSII inhibition, ion leakage, and bacterial growth in the loss-of-function *mpk3 mpk6* double mutant system after inoculation with *Pst* carrying *AvrRpm1*, *AvrB*, and *AvrRps4*. As shown in S6 Fig, PSII inhibition and ETI were all compromised in NA-PP1–, but not DMSO–, treated *MPK6SR* and *MPK3SR* plants. These results suggest that MPK3/MPK6 are essential for both CNL- and TNL-type NLR-mediated PSII inhibition and ETI.

Prolonged, but not transient, MAPK activation induces photosynthetic inhibition (Fig 3B). As a result, we examined whether long-lasting MAPK activation is essential for ETI. Wild-type *MPK6SR* and *MPK3SR* plants infiltrated with *Pst-AvrRpt2* were treated with NA-PP1 to inhibit MAPK activity at different times after inoculation. As shown in Fig 6D, NA-PP1 treatment at 12 hpi could still compromise RPS2-mediated ETI, demonstrating that short-term MAPK activation was not sufficient to confer efficient ETI.

### Photosynthetic inhibition is essential to ETI

**Fig 7:**
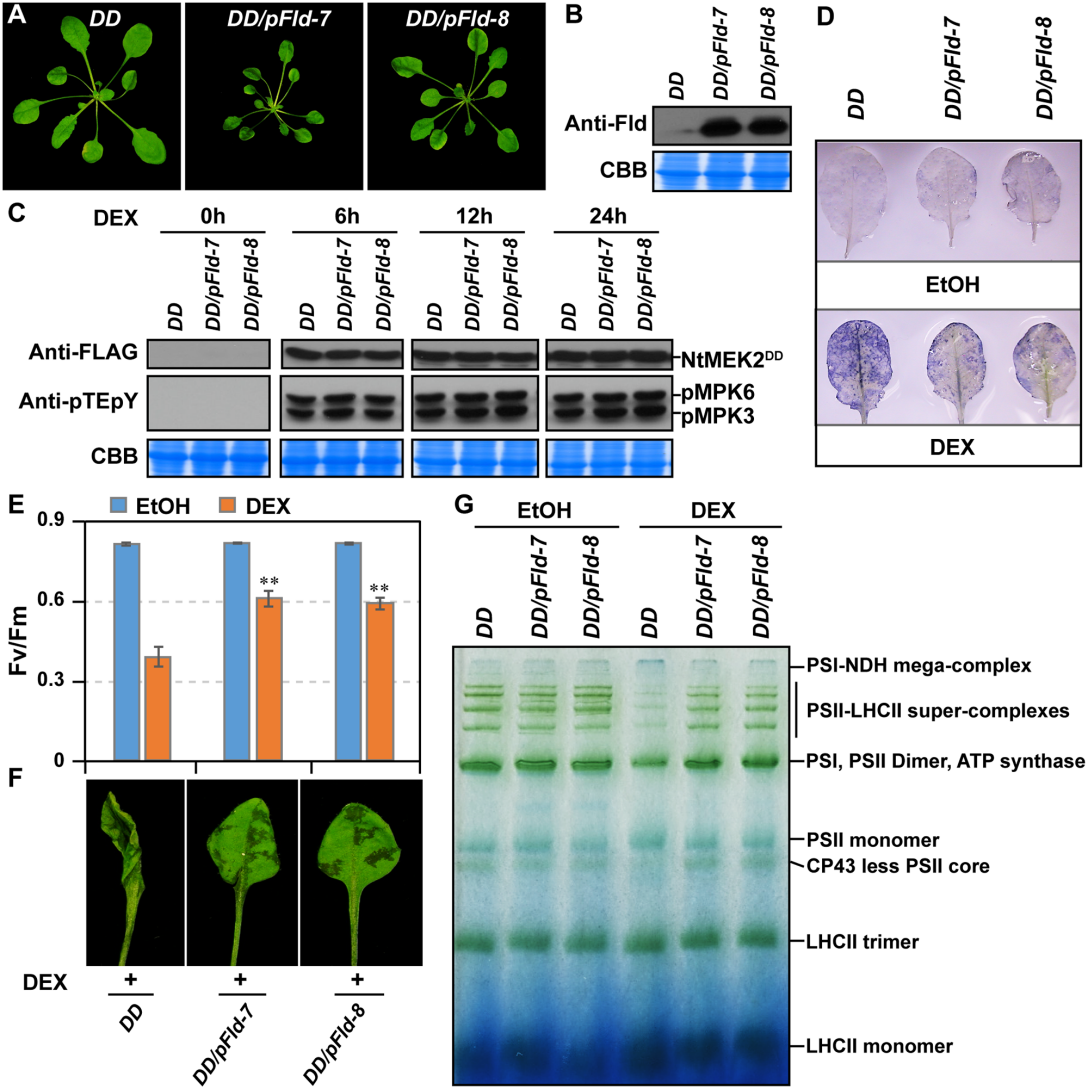
Expression of pFld impairs MPK3/MPK6 activation-induced ROS accumulation, PSII inhibition, and HR-like cell death. (**A**) Phenotype of 4-wk-old *pFld* transgenic plants in *DD* background. (**B**) Immunoblot analysis of pFld expression using anti-Fld antibody. (**C**) Normal activation of MPK3/MPK6 in *pFld* transgenic plants. Soil-grown plants were spray treated with 15 μM DEX for indicated periods of time. *DD* expression and activation of MPK3/MPK6 activation were detected by using anti-FLAG and anti-pTEpY, respectively. (**D**) Overexpression of *pFld* suppresses MPK3/MPK6 activation-induced ROS accumulation. Soil-grown *DD* and *DD/pFld* plants were infiltrated with EtOH or 5 μM DEX. ${\text{O}}_{2}^{•-}$ was visualized by NBT staining. (**E–G**) Overexpression of *pFld* suppresses MPK3/MPK6 activation-induced photosynthetic inhibition. Fv/Fm was measured at 24 h (E), HR-like phenotype was recorded at 30 h (F), and disassembly of photosynthetic complexes at 24 h was visualized by BN-PAGE (G). Samples equivalent to 8 μg of chlorophyll were loaded. Values are means ± SD, *n* = 6, **P ≤ 0.001. The numerical values used to construct panel E can be found in S1 Data. See also S7 Fig. BN-PAGE, blue native polyacrylamide gel electrophoresis; CBB, Coomassie brilliant blue; CP43, photosystem II chlorophyll protein of 43 kDa; *DD*, *GVG-NtMEK2*^*DD*^; DEX, dexamethasone; EtOH, ethanol; FLAG, an octapeptide; Fld, flavodoxin; HR, hypersensitive response; LHCII, light-harvesting complex II; MPK, mitogen-activated protein kinase; NtMEK2^DD^, constitutively activated NtMEK2; NBT, nitroblue tetrazolium; NDH, NADH dehydrogenase-like; ${\text{O}}_{2}^{•-}$, superoxide; *pFld*, plastid-targeted cyanobacterial flavodoxin; pMPK, phosphorylated MPK; PSI, photosystem I; PSII, photosystem II; pTEpY, dually phosphorylated Thr/Glu/Tyr peptide; ROS, reactive oxygen species.

To provide genetic evidence to support the importance of photosynthetic inhibition in ETI, we expressed a plastid-targeted cyanobacterial flavodoxin (pFld) in *DD* and *GVG-AvrRpt2* plants. Flowering plants do not have flavodoxin [78], and ectopically expressing a cyanobacterial flavodoxin in tobacco confers broad stress tolerance [79,80]. We found that overexpression of pFld in *DD* and *GVG-AvrRpt2* background caused growth retardation (Fig 7A and 7B, and S7 Fig). This is likely a result of the lower efficiency of flavodoxin as an electron carrier in comparison to ferredoxin [78,81]. Nonetheless, we observed that PSII inhibition induced by MPK3/MPK6 activation was impaired in pFld-overexpressing plants. Two independent pFld expression lines were used. Neither line, the induction of DD protein nor the activation of MPK3/MPK6 after DEX treatment, was affected by the overexpression of pFld (Fig 7C). As shown in Fig 7D, expression of pFld impaired MPK3/MPK6 activation-mediated ROS accumulation. Concomitantly, PSII inhibition, disassembly of photosynthetic complexes and HR-like cell death were all delayed (Fig 7E–7G). Expression of pFld also alleviated the photosynthetic inhibition triggered by conditional expression of *AvrRpt2* (S7 Fig).

**Fig 8:**
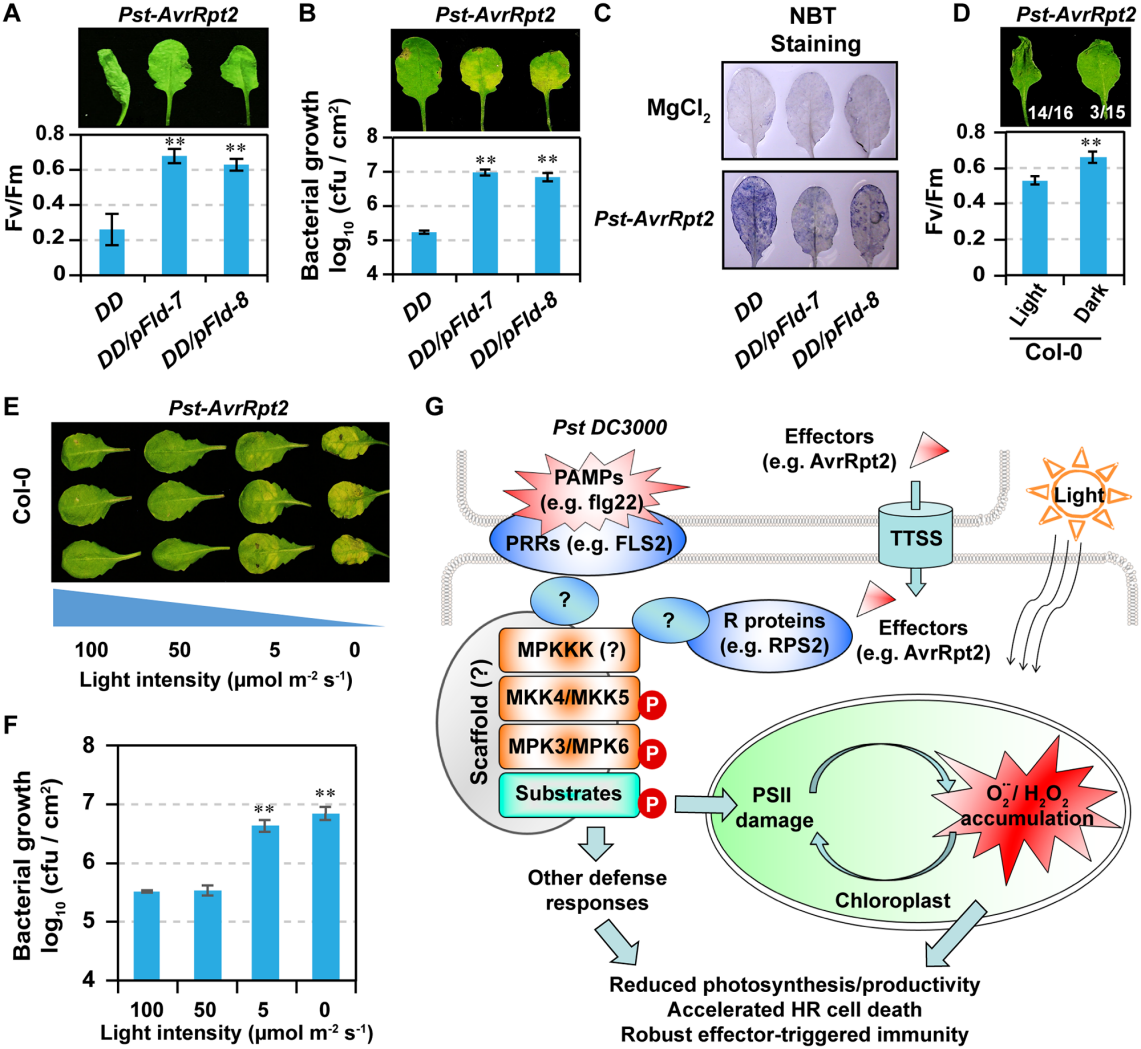
Photosynthetic inhibition and light are essential to a robust ETI. (**A**) Expression of *pFld* suppresses *Pst-AvrRpt2*–induced HR phenotype and PSII inhibition. Soil-grown *DD* and *DD/pFld* plants were infiltrated with *Pst-AvrRpt2* (OD = 0.02). Photos were taken at 24 hpi. Fv/Fm was measured at 18 hpi. Values are means ± SD, *n* = 6, **P ≤ 0.001. (**B**) Expression of *pFld* compromises disease resistance against *Pst-AvrRpt2*. Four-wk-old soil-grown *DD* and *DD/pFld* plants were infiltrated with *Pst-AvrRpt2* (OD_600_ = 0.0005). Photos were taken at 3 dpi and *Pst-AvrRpt2* growth was quantified at 2.5 dpi. Values are means ± SD, *n* = 3, **P ≤ 0.001. (**C**) Expression of *pFld* impairs *Pst-AvrRpt2*–induced ROS accumulation. Soil-grown *DD* and *DD pFld* plants were infiltrated with mock (10 mM MgCl_2_) or *Pst-AvrRpt2* (OD = 0.02). ${\text{O}}_{2}^{•-}$ was visualized by NBT staining at 6 hpi. (**D**) Light accelerates *Pst-AvrRpt2*–induced PSII inhibition and HR cell death. Four-wk-old Col-0 plants were infiltrated with *Pst-AvrRpt2* (OD = 0.02). Plants were kept in dark or under light. Photos were taken at 18 hpi and numbers are ratios of leaves with HR phenotype. Fv/Fm was measured at 12 hpi. Values are means ± SD, *n* = 7, **P ≤ 0.001. (**E** and **F**) Light is essential to plant resistance against *Pst-AvrRpt2*. Four-wk-old Col-0 plants were infiltrated with *Pst-AvrRpt2* (OD = 0.0005). After infiltration, plants were kept under light for 2 h to allow the evaporation of liquid, and then kept in dark or under different light intensities, as indicated. Photos were taken and *Pst-AvrRpt2* growth was determined at 3 dpi. Values are means ± SD, *n* = 3, **P ≤ 0.001. The numerical values used to construct panels A, B, D, and F can be found in S1 Data. (**G**) Schematic model of MPK3/MPK6 activation-induced PSII inhibition, ROS accumulation in chloroplasts, and the robustness of ETI. *AvrRpt2*, avirulence effector recognized by RPS2; Col-0, Columbia-0; *DD*, *GVG-NtMEK2*^*DD*^; dpi, days post inoculation; ETI, effector-triggered immunity; flg22, a 22 amino acids flagellin fragment; FLS2, flagellin-sensitive 2; hpi, hours post inoculation; HR, hypersensitive response; MPK, mitogen-activated protein kinase; NBT, nitroblue tetrazolium; OD, optical density; *${\text{O}}_{2}^{•-}$*, superoxide; PAMP, pathogen/microbe-associated molecular pattern; *pFld*, plastid-targeted cyanobacterial flavodoxin; PRR, pattern recognition receptor; *Pst*, *Pseudomonas syringae* pv *tomato*; PSII, photosystem II; ROS, reactive oxygen species; RPS2, Resistance to *Pseudomonas syringae* 2; TTSS, type III secretion system.

To test whether the inhibition of photosynthetic activities in chloroplasts is required for the robustness of ETI, we infiltrated *DD*, *DD*/*pFld*-*7*, and *DD*/*pFld-8* with *Pst-AvrRpt2*. In the absence of DEX, *DD* transgene is not expressed, and these plants can be treated as wild-type control and *pFId* transgenic plants, respectively. PSII inhibition induced by *Pst-AvrRpt2* was greatly delayed in *DD*/*pFld*-*7* and *DD*/*pFld-8* plants, which was associated with the inhibition of HR cell death (Fig 8A), a compromised resistance (Fig 8B), impaired ROS accumulation (Fig 8C), and delayed disassembly of photosynthetic complexes (S8 Fig). The elevated *Pst-AvrRpt2* growth led to chlorosis, a susceptible phenotype (Fig 8B). These results strongly suggest that inhibition of photosynthetic activity is essential to ETI. This notion is further supported by the observation that HR cell death and PSII inactivation is delayed in dark (Fig 7D), in which no ROS accumulation in chloroplasts was observed (Fig 2G and 2H and S5 Fig). In addition, disease resistance to *Pst-AvrRpt2* was also greatly compromised in dark or under low light, and *Pst-AvrRpt2* grew to higher titers and caused chlorosis, symptoms of susceptibility (Fig 8E and 8F). Altogether, these results suggest that light-dependent ROS accumulation in chloroplasts is an important part of ETI.

## Discussion

Decrease in plant photosynthetic activity and global down-regulation of photosynthetic genes have long been associated with plants under biotic stresses [44–50]. However, it is unclear whether this is a reflection of deterioration of plant health or an active part of plant immunity. Recently, we reported the essential role of MPK3/MPK6 in plant PTI [38]. MPK3 and MPK6 are also activated during ETI [24,25]. However, genetic evidence demonstrating the requirement of MPK3/MPK6 in ETI is still lacking. In this report, we demonstrate that gain-of-function activation of MPK3/MPK6 in *Arabidopsis* is sufficient to induce active inhibition of photosynthesis and light-dependent ROS accumulation in chloroplasts, two processes that mutually enhance each other under light. Loss-of-function data revealed that MPK3 and MPK6 are essential to effector-triggered photosynthetic inhibition and ROS accumulation in chloroplasts, and eventually ETI. This study highlights the important role of MPK3/MPK6-mediated photosynthetic inhibition and ROS accumulation in chloroplasts during ETI, which can explain why plants are more resistant under light than in dark. We propose that active photosynthetic inhibition mediated by the MPK3/MPK6 pathway is one of the key immune responses downstream of NLR activation and contributes to a robust ETI (Fig 8G).

### MPK3/MPK6 activation induces light-independent photosynthetic inhibition and light-dependent ROS accumulation in chloroplasts

MPK3/MPK6 activation-induced HR-like cell death and ROS accumulation in chloroplasts are light dependent (Fig 2G–2I). This is also true in a tobacco system [32]. However, MPK3/MPK6 activation-induced PSII inhibition can be independent of light (Figs 2A–2C and 3C). We also noticed that PSII inhibition was slower in the absence of light, which could be a result of the lack of ROS generation (Fig 2G and 2H). ROS are known to play an important role in accelerating PSII inhibition by oxidative damage of PSII proteins [82–84]. Under light, MPK3/MPK6 activation-induced PSII inactivation and ROS accumulation in chloroplasts can form a positive feed-forward loop to accelerate the PSII inhibition. Nonetheless, MPK3/MPK6 activation-induced photosynthetic inhibition can occur in the absence of light and be independent of chloroplastic ROS accumulation.

### MPK3/MPK6-mediated inhibition of photosynthesis is an important part of both CNL- and TNL-type NLR-mediated ETI

Photosynthetic inhibition is a well-documented phenomenon in plants challenged by pathogens [46–50,85–88]. However, it was not clear whether photosynthetic inhibition is a programmed part of immune response or merely a side effect caused by pathogen infection. In this study, we provided several lines of evidence suggesting that photosynthetic inhibition is an active defense response and an important part of ETI. First of all, AvrRpt2-induced photosynthetic inhibition requires its immune receptor, RPS2, demonstrating that photosynthetic inhibition is an event downstream of NLR activation. Secondly, prolonged activation of MPK3/MPK6, an event downstream of NLR activation in ETI [24], induces photosynthetic inhibition. Thirdly, ETI and MAPK signaling–mediated photosynthetic inhibition facilitate ROS accumulation in chloroplasts, which is essential to ETI. Previous studies demonstrate that light is essential for virus-induced HR [32,89]. In this study, we also found an essential role of light in *Pst-AvrRpt2*–induced HR and plant resistance against *Pst-AvrRpt2* (Fig 8D–8F). Thus, photosynthetic inhibition during ETI is actively regulated and is part of the immune response that enhances resistance.

We found that *Pst-AvrRps4* also induces prolonged MAPK activation (Fig 4G), although AvrRps4 is sensed by RPS4/RRS1, a TNL-type NLR, which was thought to function in nuclei mainly through transcriptional reprogramming [12]. The requirement of MPK3/MPK6 in both CNL- and TNL-mediated ETI raises a question of how NLR activation leads to prolonged MPK3/MPK6 activation. MPK4 is guarded by CNL-type R protein, suppressor of *mkk1 mkk2* (SUMM2), which monitors the phosphorylation status of MPK4 substrates, including MAP kinase kinase kinase 2 (MEKK2), mRNA de-capping protein PAT1, and calmodulin binding receptor-like cytoplasmic kinase 3 (CRCK3) [90–93]. It remains to be determined whether MPK3 and MPK6 are also protected by CNL- or TNL-type R proteins. In PTI, MPK3/MPK6 activation after PAMP perception by PRRs can be mediated by receptor-like cytoplasmic kinases (RLCKs), which are similar to RLKs but lack an extracellular domain. *Arabidopsis* RLCK PBS1-like 27 (PBL27) and rice (*Oryza sativa*) OsRLCK85 were demonstrated to connect chitin perception to MPK3/MPK6 activation [94,95]. It will be interesting to identify the proteins that connect NLRs to MPK3/MPK6, which may reveal the mechanism(s) underlying the prolonged activation of MPK3/MPK6 during ETI.

### MPK3/MPK6 cascade regulates the trade-off between growth and defense in plant immunity

The concept of a trade-off between growth and defense has been proposed for many decades [96]. Our current knowledge on the growth–defense trade-off mainly stems from antagonistic cross talk among hormones that promote defense and that promote growth, such as SA-auxin, SA-brassinosteroid (BR), SA-gibberellic acid (GA), jasmonic acid (JA)-Auxin, JA-BR, and JA-GA [97–99]. However, how a plant integrates multiple internal and external stimuli to shift the balance between growth and defense remains poorly understood. It is also unclear why these two events are coupled together most of the time. We showed in this report that both events are regulated by the same MAPK signaling pathway. MPK3/MPK6 activation globally down-regulates photosynthetic genes and, in the meantime, up-regulates numerous defense-related genes (Fig 1A–1C), suggesting that MAPK signaling plays important roles in orchestrating growth and defense in plant immunity. Consistent with the down-regulation of photosynthetic genes, we did observe decreases in CO_2_ fixation [32] and photosynthetic inhibition (Fig 2A–2C). Both would have negative impacts on normal plant growth and development. In the meantime, up-regulation of defense genes by the MPK3/MPK6 cascade leads to an increased biosynthesis of defense-related secondary metabolites such as camalexin [53] and indole glucosinolate derivatives [56]. Considering that multiple developmental and environmental signals converge at the MPK3/MPK6 cascade [28,100], we propose that the MPK3/MPK6 cascade is a key hub in orchestrating the trade-off between growth and defense.

MPK3/MPK6 activation-induced photosynthetic inhibition, as well as its associated ROS accumulation and HR cell death, contribute positively to the robust ETI. Regulation of photosynthetic inhibition by an active signaling cascade demonstrates that the inhibition of photosynthesis is an active defense response in plant immunity triggered by effectors, not a passive consequence of the deterioration of plant fitness caused by pathogen infection. It also reveals a potential mechanism underlying the growth–defense trade-off during plant immunity. Plant ETI is a stronger and more robust form of immune response in comparison to PTI [3,7,8]. In such a case, a robust defense, but not growth, is of high priority. Long-lasting activation of MPK3/MPK6 triggered by pathogen effectors contributes to the robustness of ETI (Fig 8G). It is worth noting that PTI, a weaker form of plant immunity, induces only transient MAPK activation and does not cause photosynthetic inhibition (Fig 3B and S4 Fig), indicating that photosynthetic activities are differently regulated during different forms of immune responses by the same MAPK signaling pathway, depending on its activation kinetics.

## Materials and methods

### Plant growth

Soil-grown *Arabidopsis* plants were maintained at 22 °C and about 70% relative humidity in a growth chamber with a 10 h day/14 h night cycle (100 μmol m^−2^ s^−1^). For plants grown in liquid medium, seeds were surface sterilized. After stratification at 4 °C for 3–5 d, seeds were sown in petri dishes with liquid half-strength Murashige and Skoog medium and grown in a growth chamber at 22 °C with continuous light (70 μmol m^−2^ s^−1^). Six-d-old seedlings were transferred to 20-mL GC vials with 6 mL of liquid half-strength MS medium (10 seedlings per vial) and maintained under the same growth conditions [25]. Col-0 ecotype was used as the wild type. Mutant alleles and transgenic lines of *mpk3-1* (Salk_151594), *mpk6-2* (Salk_073907), *DD* (*GVG-NtMEK2*^*DD*^), *AvrRpt2/RPS2* (*GVG-AvrRpt2* in Col-0 background), *AvrRps2/rps2* (*GVG-AvrRpt2* in *rps2-101C* background), *MPK6SR* (*mpk3 mpk6 P*_*MPK6*_:*MPK6*^*YG*^, Line #58), and *MPK3SR* (*mpk3 mpk6 P*_*MPK3*_:*MPK3*^*TG*^, Line #64) were reported previously [52,65,101].

### Generation of transgenic lines

For generation of *DD/pFld* and *GVG-AvrRpt2/pFld* plants, the coding sequence of *Fld* from cyanobacterium *Anabaena sp*. PCC 7119 [102] was first optimized to codons preferred in *Arabidopsis* using OptimumGene algorithm (Genscript) (S9 Fig). After introducing *Nde* I and *Spe* I enzyme digestion sites, the *Nde* I*-Fld-Spe* I fragment was directly synthesized into *pUC57* vector, and then the *Nde I-Fld-Spe I* fragment was subcloned into *pBluescript* (*pBS*) vectors with RbcS signal peptide sequence to generate *pBS-RbcS-Fld*. The *pBS-RbcS-Fld* was cut with *Xho* I and *Spe* I and subcloned into *pBID* vector to generate *pBID-RbcS-Fld* constructs. The *pBID-RbcS-Fld* construct was then transformed to *Agrobacterium tumefaciens* GV3101. Finally, the *A*. *tumefaciens* GV3101 containing *pBID-RbcS-Fld* was used to transform *DD* and *GVG-AvrRpt2/RPS2* plants, respectively. Single insertion lines were selected and the expression of Fld was confirmed by immunoblot. F3 homozygous *DD/pFld* and *GVG-AvrRpt2/pFld* plants were used for experiments.

### Chlorophyll fluorescence measurement

The O-J-I-P curve was measured by using Dual-PAM chlorophyll fluorometer (Walz, Germany) with a built-in fast kinetic protocol. Other chlorophyll fluorescence parameters were measured with the Maxi-version of Imaging-PAM chlorophyll fluorometer (Walz, Germany) or FMS2 (Hansatech, United Kingdom). F_o_ (minimum fluorescence of dark adapted leaves) was measured using weak light (<1 μmol m^−2^ s^−1^) at a low frequency (2 Hz). For measuring F_m_ (maximum fluorescence yield of dark-adapted leaves), dark-adapted leaves were exposed to a PPFD of approximately 2,700 μmol m^−2^ s^−1^. When performing induction kinetics measurements, the intensity of actinic light was set to 110 μmol m^−2^ s^−1^. For NPQ induction analysis, the intensity of actinic light was set to 610 μmol m^−2^ s^−1^. The interval for measuring Fm’ (maximum fluorescence yield of light adapted leaves) was 20 s. Maximal PSII quantum yield (F_v_/F_m_) was calculated with (F_m_-F_o_)/F_m_; effective quantum yield of PSII (Y(II)) with (F_m'_−F)/F_m'_; qL, the parameter estimating the open PSII centers based on a lake model, with (F_m_/F–F_m_/F_m'_) / (F_m_/F_o_ –1); and NPQ, the nonphotochemical quenching parameter describing the regulated quenching of excessive energy, with (F_m_−F_m'_)/F_m'_.

### ROS staining

In vivo H_2_O_2_ generation in plants was detected by using DAB as described previously [32]. Twelve-d-old *Arabidopsis* seedlings after treatment were submerged into a solution containing 1 mg/mL DAB (pH 5.5) for 2 h under growth light. Oxidation of DAB leads to its polymerization and deposition at the site of ROS generation. The seedlings were then boiled in ethanol for 10 min to remove chlorophyll. H_2_O_2_ production is visualized as a reddish-brown coloration. In vivo ${\text{O}}_{2}^{•-}$ production was monitored by NBT staining as described previously [32]. Twelve-d-old *Arabidopsis* seedlings after treatment were submerged into 10 mM potassium phosphate buffer (pH 7.8) containing 1 mg/mL NBT and 10 mM NaN_3_. To avoid overstaining, the seedlings were stained in dark for 30 min. The seedlings were then boiled in ethanol for 10 min to remove chlorophyll. Reduced NBT was visualized as a dark blue-colored formazam deposit. Single layer mesophyll cells were prepared according to [103] for visualization of H_2_O_2_ and ${\text{O}}_{2}^{•-}$ accumulation in mesophyll cells. Seedlings stained with DAB and NBT were fixed in 3.5% glutaraldehyde for 1 h and then softened with 0.1 M EDTA, pH 9.0, for 2 h at 55 °C. A leaf sample (about 1 mm^2^) was placed on a glass slide and covered with a cover slide. The leaf sample was stretched into a single cell layer by lightly tapping with the eraser of a pencil. H_2_O_2_ and ${\text{O}}_{2}^{•-}$ accumulation in chloroplasts were imaged with a microscope equipped with a digital camera.

### Pathogen and ion leakage assay

HR assay was performed as described previously [104]. For HR assay in Col-0, *DD*, and *DD/pFld* plants, 4-wk-old plants were infiltrated with *Pst-AvrRpt2* (OD_600_ = 0.02). For Col-0, *MPK6SR*, and *MPK3SR*, 4-wk-old plants were first sprayed with 10 μM NA-PP1 or DMSO (mock) 2 h before *Pst-AvrRpt2* (OD_600_ = 0.02) infiltration. After infiltration, plants were kept under a growth light and HR phenotype was detected at 18 hpi. The disease resistance assay was carried out as previously described [25]. For the resistance assay in *DD* and *DD/pFld*, 4-wk-old plants were infiltrated with *Pst-AvrRpt2* (OD_600_ = 0.0005). For Col-0, *MPK6SR*, and *MPK3SR*, 4-wk-old plants were first sprayed with 10 μM NA-PP1 or DMSO (mock) 2 h before or at indicated times for *Pst-AvrRpt2* (OD_600_ = 0.0005), *Pst-AvrRpm1* (OD_600_ = 0.0005), *Pst-AvrB* (OD_600_ = 0.0005), and *Pst-AvrRps4* (OD_600_ = 0.0005) infiltration. Plants were maintained at 22 °C in a growth chamber with a 10 h day/14 h night cycle (100 μmol m^−2^ s^−1^). Normally, for Col-0, *MPK6SR*, and *MPK3SR* were sprayed with 10 μM NA-PP1 or DMSO (mock) again at 1.5 dpi if not specified. Bacterial growth was quantified with 12 leaves (usually the fifth and sixth leaves) from 6 independent plants at 2.5 or 3 dpi.

Ion leakage assay was performed as previously described [105]. Plants were first sprayed with 10 μM NA-PP1 or DMSO (mock). Leaf discs (7 mm in diameter) were punched out and then vacuum infiltrated with *Pst-AvrRpt2* (OD_600_ = 0.02), *Pst-AvrRpm1* (OD_600_ = 0.02), *Pst-AvrB* (OD_600_ = 0.02), or *Pst-AvrRps4* (OD_600_ = 0.02). Leaf discs were then transferred to a 20-mL GC vial containing 10 mL ddH_2_O with 2 μM NA-PP1 or DMSO (mock). Conductivity was measured by using a conductivity meter.

### BN-PAGE and two-dimensional SDS-PAGE

Photosynthetic complexes were separated by BN-PAGE according to the modified protocol [106]. Twelve-d-old *Arabidopsis* seedlings grown in liquid medium were treated with 5 μM DEX for indicated time points. Seedlings (about 1.5 g) were ground in 10 mL thylakoid extraction buffer (50 mM HEPES/KOH, pH 7.5, 330 mM sorbitol, 2 mM EDTA, 1 mM MgCl_2_, 5 mM ascorbate, 0.05% [w/v] BSA, and 10 mM NaF) and filtered through 4 layers of KimWipes. After centrifugation for 5 min at 4 °C, 2,500*g*, the pellet was successively washed with washing buffer I (50 mM HEPES/KOH, pH 7.5, 5 mM sorbitol, and 10 mM NaF) and washing buffer II (50 mM HEPES/KOH, pH 7.5, 100 mM sorbitol, 10 mM MgCl_2_, and 10 mM NaF) at 2,500*g* for 5 min at 4 °C. Chlorophyll was extracted in 80% (v/v) buffered acetone (2.5 mM HEPES/NaOH, pH 7.5) and the content of chlorophyll was determined according to the equation: Chlorophyll (μg/mL) = 20.2A_645_ + 8A_663_. The thylakoid membrane was washed twice with a buffer (25 mM Bis Tris-HCl, pH 7.0, 20% [v/v] glycerol) at 12,000*g* for 5 min at 4 °C. Then, the thylakoids were solubilized in solubilization buffer (25 mM Bis Tris-HCl, pH 7.0, 20% [v/v] glycerol and 1% n-dodecyl-b-maltoside) for 10 min on ice. After solubilization, the samples were centrifuged at 12,000*g* for 10 min at 4 °C to remove insoluble ingredients. The supernatant was transferred to a new tube, and 1/10 volume of 10× native sample buffer (100 mM Bis Tris-HCl, pH 7.0, 0.5 M 6-amino-caproic acid, 30% [v/v] glycerol, and 0.5% [v/v] brilliant blue G-250) were added. Thylakoid membranes equivalent to 8 μg chlorophyll content were loaded to a 5%–13.5% gradient native PAGE gel and run at voltages of 50, 75, 100, 125, 150, 175, 200, 225, and 250 V, each for 0.5 h. After the first dimensional separation, strips were excised and incubated with 138 mM Tris/HCl, pH 6.8, 6 M urea, 22.2% (v/v) glycerol, 4.3% (w/v) SDS, and 5% (v/v) 2-mercaptoethanol for 1 h at room temperature. Then, the strips were loaded to a 15% (w/v) SDS-PAGE gel containing 6 M urea. Proteins on the 2D gels were visualized by staining with brilliant blue G-250.

### Protein extraction and immunoblot analysis

Protein extraction and immunoblot were carried out as previously described [32]. Total protein was extracted from leaf tissue by grinding with small plastic pestles in extraction buffer (100 mM HEPES, pH 7.5, 5 mM EDTA, 5 mM EGTA, 10 mM DTT, 10 mM Na_3_VO_4_, 10 mM NaF, 50 mM b-glycerophosphate, 1 mM phenylmethylsulfonyl fluoride, 5 μg/mL antipain, 5 μg/mL aprotinin, 5 μg/mL leupeptin, and 10% [v/v] glycerol). Thylakoid proteins for immunoblot were prepared by adding 1/4 volume of 4× SDS sample buffer (250 mM Tris/HCl, pH 6.8, 40% [v/v] glycerol, 4% [w/v] SDS, and 0.1% [w/v] bromophenol blue) to solubilized thylakoid preparations and boiled for 2 min. NtMEK2^DD^ expression was detected by using anti-FLAG (Sigma, F1804, dilution 1:10,000). Activation of MPK3 and MPK6 was detected by using anti-pTEpY (Cell signaling, dilution 1:4,000). Expression of Fld was detected by anti-Fld (a gift from Dr. Donald A. Bryant, Pennsylvania State University, dilution 1:3,000). Anti-D1 antibody (Agrisera, AS10704, dilution 1:10,000) was used for the detection of PSII core D1 protein. The blots were incubated with horseradish peroxidase-conjugated goat-anti-mouse or goat-anti-rabbit secondary antibodies (dilution 1:10,000), and the bands were visualized using an enhanced chemiluminescence kit (Perkin Elmer) according to the manufacturer’s instructions.

### Quantitative RT-PCR analysis

Real-time quantitative PCR (qPCR) was performed as previously described [54]. Total RNA was extracted using TRizol reagent (Invitrogen). After DNase treatment, 1 μg of total RNA was used for reverse transcription. Real-time qPCR analysis was performed using an ABI 7500 real-time PCR machine (Life Technologies). *EF-1a* was used as an internal control. The primer pairs used for qPCR are listed in S2 Table.

### Illumina RNA-seq gene expression profiling

Total RNA was extracted with TRizol reagent (Invitrogen) from 12-d-old *DD* seedlings treated with 2 μM DEX for 0 and 6 h, respectively. After DNase treatment, total RNA was purified using RNA clean and concentrator Kit. RNA sequencing libraries were constructed using TruSeq RNA library preparation Kit and sequenced using the HiSeq X Ten according to the manufacturer’s instructions. Dirty raw reads were filtered out using SONPnuke software. Clean reads were mapped to the *Arabidopsis* reference genome with BWA and to reference gene sequences with Bowtie. Gene expression levels were calculated using the RPKM method (reads per kb per million reads). The raw Illumina reads generated from RNA-seq experiments were deposited at NCBI Sequence Read Archive (SRP111959).

### Quantification and statistical analysis

Statistical details of experiments are reported in figure legends. Statistical significance between groups was determined by one-way ANOVA comparison; *p*-values were indicated in figure legends. Each experiment was performed at least twice, with similar results.

## Supporting information

S1 Fig Activation of MPK3/MPK6 causes disassembly of photosynthetic complexes, related to Fig 2. Activation of MPK3/MPK6 causes disassembly of PSII-LHCII super-complexes and accumulation of the intermediate CP43-less PSII core complex. Twelve-d-old *DD* plants grown in liquid medium were treated with 5 μM DEX for indicated periods of time. Thylakoid membrane samples equivalent to 8 μg of chlorophyll content were subjected to first dimension BN-PAGE. The BN-PAGE strips were cut out. After denaturation, the samples were subjected to second dimension SDS-PAGE. Protein spots were visualized by brilliant blue G250 staining. Bands and protein spots were labeled as described previously. BN-PAGE, blue native polyacrylamide gel electrophoresis; CP43, photosystem II chlorophyll protein at 43 kDa; *DD*, *GVG-NtMEK2*^*DD*^; DEX, dexamethasone; LHCII, light-harvesting complex II; mc, mega-complex; MPK, mitogen-activated protein kinase; PSII, photosystem II; sc, super-complex; SDS-PAGE, sodium dodecyl sulfate-PAGE.
(TIF)

S2 FigActivation of MPK3/MPK6 causes ROS accumulation, related to Fig 2.Activation of MPK3/MPK6 leads to increases in H_2_O_2_ (**A**) and ${\text{O}}_{2}^{•-}$ (**B**) levels in whole seedlings. *DD* plants grown in liquid medium were treated with EtOH or 5 μM DEX and were kept under light for indicated periods of time. H_2_O_2_ and ${\text{O}}_{2}^{•-}$ were visualized by DAB and NBT staining, respectively. DAB, 3,3′-diaminobenzidine; *DD*, *GVG-NtMEK2*^*DD*^; DEX, dexamethasone; EtOH, ethanol; MPK, mitogen-activated protein kinase; NBT, nitroblue tetrazolium; ROS, reactive oxygen species.(TIF)

S3 Fig Transient activation of MPK3/MPK6 after flg22 treatment does not induce a decrease in PSII core D1 protein, related to Fig 3. Twelve-d-old *DD* and Col-0 plants grown in liquid medium were treated with 5 μM DEX and 200 nM flg22 for indicated times, respectively. Thylakoid membranes were isolated and solubilized with 1% dodecyl maltoside. Samples equivalent to 8 μg of chlorophyll were loaded to a BN-PAGE. For detection of D1 abundance in different complexes, samples equivalent to 2 μg of chlorophyll were loaded to a BN-PAGE. After transferring to a PVDF membrane, anti-D1 was used to detect D1 abundance. BN-PAGE, blue native polyacrylamide gel electrophoresis; Col-0, Columbia-0; DEX, dexamethasone; flg22, a 22 amino acids flagellin fragment; MPK, mitogen-activated protein kinase; PSII, photosystem II; PVDF, polyvinylidene fluoride.
(TIF)

S4 Fig ETI is associated with photosynthetic inhibition, related to Fig 4. (**A** and **B**) Photosynthetic parameters are differentially affected by different *Pst* strains. Four-wk-old Col-0 plants were infiltrated with 10 mM MgCl_2_ (mock), *Pst-EV*, *Pst-AvrRpt2*, or *Pst-hrcC*^−^ (OD_600_ = 0.2). The plants were covered with a transparent lid (100% relative humidity) and kept under light. Fv/Fm and Y(II) were measured at the indicated time. Values are means ± SD, *n* = 8. (**C**) Flg22-triggered PTI does not induce photosynthetic inhibition. Four-wk-old Col-0 plants were infiltrated with 10 mM MgCl_2_ (mock) or 50 nM flg22. Photosynthetic parameters were measured at 24 hpi. Values are means ± SD, *n* = 8. (**D** and **E**) AvrRpm1-triggered ETI also induces photosynthetic inhibition. Four-wk-old Col-0 plants were infiltrated with 10 mM MgCl_2_ (mock), *P*. *fluorescens-EV* (*Pf0-1-EV*), or *P*. *fluorescens-AvrRpm1* (*Pf0-1-AvrRpm1*) (OD_600_ = 0.02). Fv/Fm and Y(II) were measured at indicated time points. Values are means ± SD, *n* = 8. The numerical values used to construct panels A–E can be found in S1 Data. AvrRpm1, avirulence effector recognized by RPM1; *AvrRpt2*, avirulence effector recognized by RPS2; Col-0, Columbia-0; ETI, effector-triggered immunity; *EV*, empty vector; flg22, a 22 amino acids flagellin fragment; hpi, hours post inoculation; *hrcC*^−^, outer membrane type III secretion protein HrcC mutant; OD, optical density; *Pf0-1-AvrRpm1*, *P*. *fluorescens-AvrRpm1*; *Pf0-1-EV*, *P*. *fluorescens-EV*; *Pst*, *Pseudomonas syringae* pv *tomato*; PTI, PAMP-triggered immunity.
(TIF)

S5 FigETI causes ROS accumulation in chloroplast, related to Fig 4.AvrRpt2-mediated ETI results in light-dependent increases in ${\text{O}}_{2}^{•-}$ and H_2_O_2_ production in chloroplasts. Twelve-d-old *GVG-AvrRpt2/RPS2* and *GVG-AvrRpt2/rps2* plants were treated with DEX (5 μM) or EtOH solvent control for 8 h. ${\text{O}}_{2}^{•-}$ and H_2_O_2_ accumulation was detected by NBT (**A**) and DAB (**B**) staining, respectively. Images of ROS accumulation at whole leaf level and subcellular level were shown. AvrRpt2, avirulence effector recognized by RPS2; DAB, 3,3′-diaminobenzidine; DEX, dexamethasone; ETI, effector-triggered immunity; EtOH, ethanol; *GVG-AvrRpt2*, DEX-inducible promoter-driven *AvrRpt2*; NBT, nitroblue tetrazolium; ${\text{O}}_{2}^{•-}$, superoxide; ROS, reactive oxygen species; *RPS2*, Resistance to *Pseudomonas syringae* 2.(TIF)

S6 Fig MPK3 and MPK6 are required for both CNL and TNL NLR-mediated ETI, related to Fig 5. (**A**) Four-wk-old plants were first sprayed with DMSO or 10 μM NA-PP1 for 2 h before infiltration with *Pst-AvrRpm1* (OD_600_ = 0.0005), *Pst-AvrB* (OD_600_ = 0.0005), or *Pst-AvrRps4* (OD_600_ = 0.0005). NA-PP1 or DMSO was sprayed again at 1.5 dpi. *Pst-AvrRpt2* growth was quantified at 3 dpi. Values are means ± SD, *n* = 3, *P ≤ 0.001. (**B**) Plants pretreated with DMSO or 10 μM NA-PP1 were infiltrated with *Pst-AvrRpm1* (OD_600_ = 0.02), *Pst-AvrB* (OD_600_ = 0.02), or *Pst-AvrRps4* (OD_600_ = 0.02). Soil-grown Col-0, *MPK6SR*, and *MPK3SR* plants were first spray treated with 10 μM NA-PP1 for 2 h, and leaf discs were punched and then infiltrated with *Pst-AvrRpt2* (OD = 0.02) by vacuum. Leaf discs were then transferred to GC vials containing 2 μM NA-PP1 or DMSO. Ion leakage was measured as increase in conductivity. Values are means ± SD, *n* = 3. (**C**) Four-wk-old soil-grown Col-0, *MPK6SR*, and *MPK3SR* plants were first spray treated with NA-PP1 (10 μM) or DMSO solvent control for 2 h and then infiltrated with *Pst-AvrRpm1* (OD = 0.02), *Pst-AvrB* (OD = 0.02), and *Pst-AvrRps4* (OD = 0.02). Values are means ± SD, *n* = 8. The numerical values used to construct panels A–C can be found in S1 Data. *AvrB*, avirulence protein B; *AvrRpm1*, avirulence effector recognized by RPM1; *AvrRps4*, avirulence effector recognized by RPS4; CNL, coiled coil-nucleotide binding site-leucine rich repeat; Col-0, Columbia-0; ETI, effector-triggered immunity; GC, gas chromatography; MPK, mitogen-activated protein kinase; NA-PP1, 4-amino-1-tert-butyl-3-(1’-naphthyl)pyrazolo[3,4-d]pyrimidine; NLR, nucleotide-binding leucine-rich repeat; OD, optical density; *Pst*, *Pseudomonas syringae* pv *tomato*; TNL, toll/interleukin-1 receptor-nucleotide binding site-leucine rich repeat.
(TIF)

S7 Fig Expression of pFld impairs AvrRpt2-induced PSII inhibition, related to Fig 7. (**A**) Expression of pFld causes growth retardation. Four-wk-old soil-grown *pFld* transgenic plants in *GVG-AvrRpt2* background were shown. (**B**) Immunoblot analysis of Fld expression in *pFld* transgenic plants using anti-Fld antibody. (**C**) AvrRpt2-induced PSII inactivation is delayed in *pFld* transgenic lines. Twelve-d-old *GVG-AvrRpt2*, *GVG-AvrRpt2/pFld-4*, and *GVG-AvrRpt2/pFld-34* plants grown in liquid medium were treated with EtOH or 5 μM DEX. Fv/Fm was measured at 18 hpi. Values are means ± SD, *n* = 6, **P ≤ 0.001. The numerical values used to construct panel C can be found in S1 Data. AvrRpt2, avirulence effector recognized by RPS2; DEX, dexamethasone; EtOH, ethanol; Fld, flavodoxin; *GVG-AvrRpt2*, DEX-inducible promoter-driven *AvrRpt2*; hpi, hours post inoculation; pFld, plastid-targeted cyanobacterial flavodoxin; PSII, photosystem II.
(TIF)

S8 Fig Expression of pFld impairs AvrRpt2-induced PSII inhibition, related to Fig 8. Twelve-d-old *DD* and *DD pFld* plants grown in liquid medium were treated with *Pst-AvrRpt2* (OD = 0.02); disassembly of photosynthetic complexes at 24 hpi was visualized with BN-PAGE. Thylakoid membranes were isolated and solubilized with 1% dodecyl maltoside. Samples equivalent to 8 μg of chlorophyll were loaded to a blue native polyacrylamide gel (BN-PAGE). AvrRpt2, avirulence effector recognized by RPS2; BN-PAGE, blue native polyacrylamide gel electrophoresis; *DD*, *GVG-NtMEK2*^*DD*^; hpi, hours post inoculation; OD, optical density; pFLD; plastid-targeted cyanobacterial flavodoxin; PSII, photosystem II; *Pst*, *Pseudomonas syringae* pv *tomato*.
(TIF)

S9 Fig Original and optimized sequence for Fld. The coding sequence of *Fld* from cyanobacterium *Anabaena sp*. PCC 7119 was optimized using OptimumGene algorithm (Genscript, http://www.genscript.com). *Fld*, flavodoxin.
(TIF)

S1 Table List of MPK3/MPK6 activation-regulated genes. MPK, mitogen-activated protein kinase.
(XLSX)

S2 Table Primers used in this study. (XLSX)

S1 Data Numerical values for bar and line charts in this study. (XLSX)

## Abbreviations

*AvrB*: avirulence protein B
*AvrRpm1*: avirulence effector recognized by RPM1
*AvrRps4*: avirulence effector recognized by RPS4
*AvrRpt2*: avirulence effector recognized by RPS2
BN-PAGE: blue native-polyacrylamide gel electrophoresis
BR: brassinosteroid
CBB: Coomassie brilliant blue
CC: coiled-coil
CKI: cycline-dependent kinase inhibitor
CNL: coiled coil-nucleotide binding site-leucine rich repeat
Col-0: Columbia-0
CP43: photosystem II chlorophyll protein at 43 kDa
CRCK3: calmodulin binding receptor-like cytoplasmic kinase 3
DAB: 3,3′-diaminobenzidine
DAVID: Database for Annotation, Visualization and Integrated Discovery
*DD*: *GVG-NtMEK2*^*DD*^
DEX: dexamethasone
dpi: days post inoculation
EDS1: Enhanced Disease Susceptibility 1
ETI: effector-triggered immunity
EtOH: ethanol
ETS: effector-triggered susceptibility
Fd: ferredoxin
FLAG: an octapeptide
Fld: flavodoxin
flg22: a 22 amino acids flagellin fragment
FLS2: flagellin-sensitive 2
FNR: ferredoxin-NADP^+^ reductase
Fv/Fm: maximal PSII quantum yield
GA: gibberellic acid
GC: gas chromatography
GO: gene ontology
*GVG-AvrRpt2*: DEX-inducible promoter-driven *AvrRpt2*
H_2_O_2_: hydrogen peroxide
hpi: hours post inoculation
HR: hypersensitive response
*hrcC*^−^: mutant of outer membrane type III secretion protein HrcC
JA: jasmonic acid
LHCI: light-harvesting complex I
LHCII: light-harvesting complex II
LRR: leucine-rich repeat
MEKK2: MPK kinase kinase kinase 2
MPK: mitogen-activated protein kinase
MS: Murashige and Skoog medium
NA-PP1: 4-amino-1-tert-butyl-3-(1’-naphthyl)pyrazolo[3,4-d]pyrimidine
NB-ARC: nucleotide binding site and ARC subdomain originating in Apaf1, R proteins, and CED-4
NBS: nucleotide binding site
NBT: nitroblue tetrazolium
NDH: NADH dehydrogenase-like
NLR: nucleotide-binding leucine-rich repeat R protein
NPQ: nonphotochemical quenching
NtMEK2: *Nicotiana tabacum* MAP kinase kinase 2
${\text{O}}_{2}^{•-}$: superoxide
OD: optical density
PAMP: pathogen/microbe-associated molecular pattern
*pBS*: *pBluescript*
*Pf0-1-AvrRpm1*: *P*. *fluorescens-AvrRpm1*
*Pf0-1-EV*: *P*. *fluorescens-EV*
pFld: plastid-targeted cyanobacterial flavodoxin
pMPK: phosphorylated MPK
PQ: plastoquinol
PRR: pattern recognition receptor
PSI: photosystem I
PSII: photosystem II
*Pst*: *Pseudomonas syringae* pv *tomato*
*Pst-EV*: *Pseudomonas syringae* pv. *tomato DC3000* carrying empty vector
pTEpY: dually phosphorylated Thr/Glu/Tyr peptide
PTI: PAMP-triggered immunity
PVDF: polyvinylidene fluoride
qPCR: quantitative PCR
RLCK: receptor-like cytoplasmic kinase
RLK: receptor-like protein kinase
RLP: receptor-like protein
RNA-seq: RNA sequencing
ROS: reactive oxygen species
RPM1: Resistance to *Pseudomonas syringae* pv *maculicola* 1
RPS2: Resistance to *Pseudomonas syringae* 2
RPS4: Resistance to *Pseudomonas syringae* 4
RPS6: Resistance to *Pseudomonas syringae* 6
RRS1: Resistance to *Ralstonia solanacearum* 1
RT-PCR: reverse transcription-polymerase chain reaction
SA: salicylic acid
SDS-PAGE: sodium dodecyl sulfate (SDS)-PAGE
SIPK: SA-induced protein kinase
SUMM2: suppressor of *mkk1 mkk2*
TIR: toll/interleukin-1 receptor
TNL: toll/interleukin-1 receptor-nucleotide binding site-leucine rich repeat
TTSS: type III secretion system
WIPK: wound-induced protein kinase
Y(II): PSII operating efficiency
