# Supplementary figures and images for: Active photosynthetic inhibition mediated by MPK3/MPK6 is critical to effector-triggered immunity
Source: PLoS Biol. 2018 May 3;16(5):e2004122. doi: 10.1371/journal.pbio.2004122 (PMC5953503; doi:10.1371/journal.pbio.2004122)

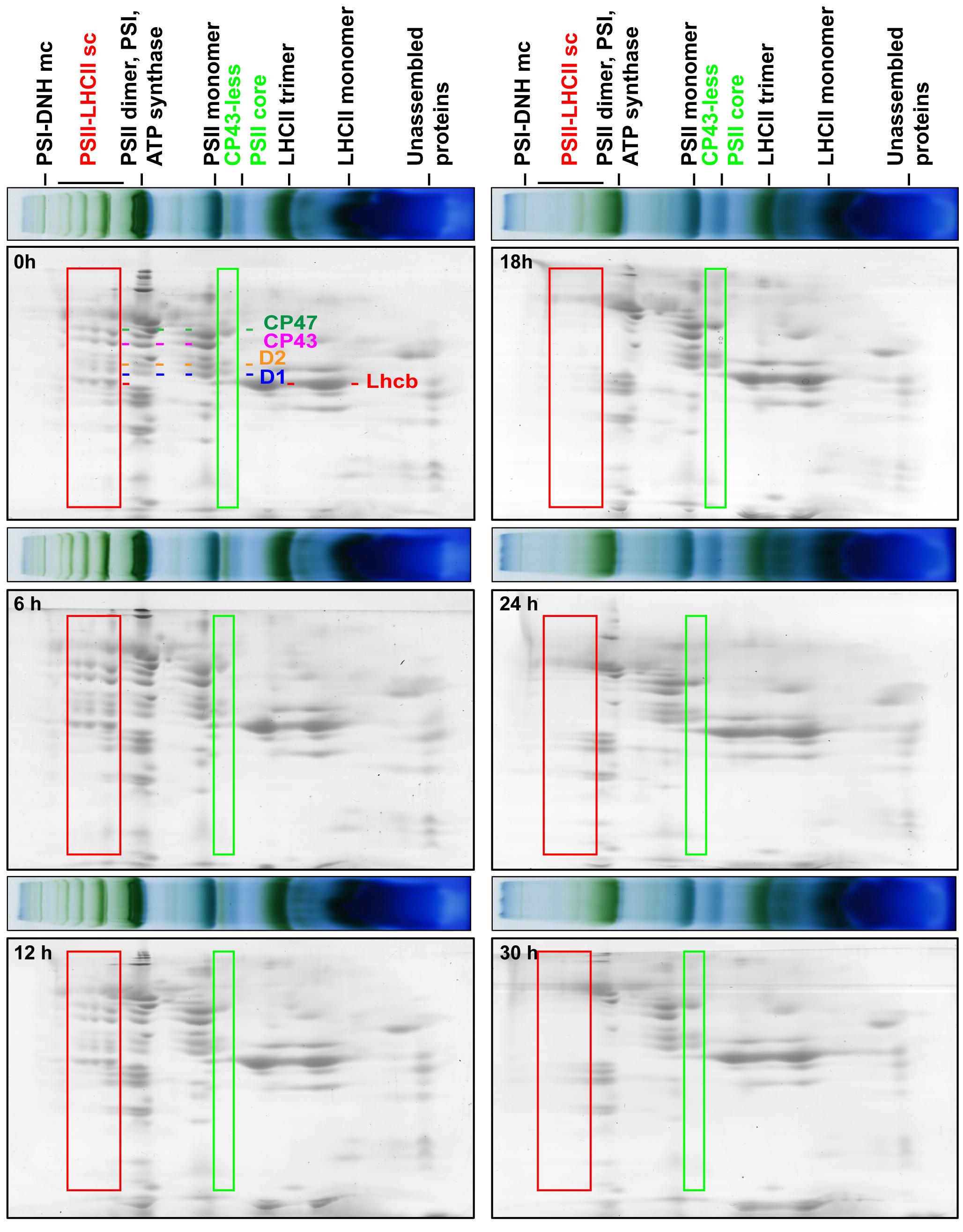

Supplement: S1 Fig — Activation of MPK3/MPK6 causes disassembly of PSII-LHCII super-complexes and accumulation of the intermediate CP43-less PSII core complex. Twelve-d-old DD plants grown in liquid medium were treated with 5 μM DEX for indicated periods of time. Thylakoid membrane samples equivalent to 8 μg of chlorophyll content were subjected to first dimension BN-PAGE. The BN-PAGE strips were cut out. After denaturation, the samples were subjected to second dimension SDS-PAGE. Protein spots were visualized by brilliant blue G250 staining. Bands and protein spots were labeled as described previously. BN-PAGE, blue native polyacrylamide gel electrophoresis; CP43, photosystem II chlorophyll protein at 43 kDa; DD, GVG-NtMEK2DD; DEX, dexamethasone; LHCII, light-harvesting complex II; mc, mega-complex; MPK, mitogen-activated protein kinase; PSII, photosystem II; sc, super-complex; SDS-PAGE, sodium dodecyl sulfate-PAGE. (TIF) [file pbio.2004122.s001.tif]

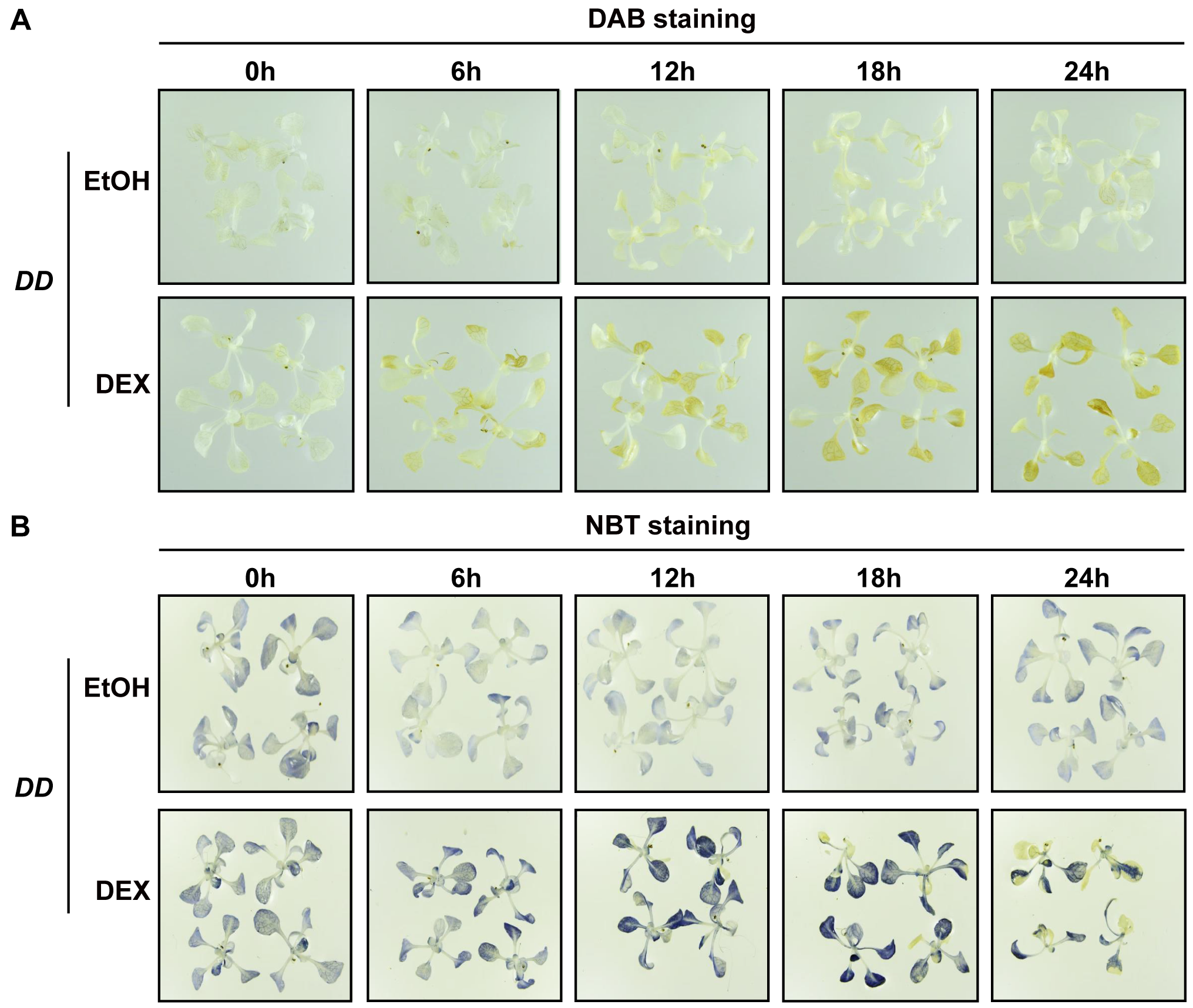

Supplement: S2 Fig — Activation of MPK3/MPK6 leads to increases in H2O2 (A) and O2•− (B) levels in whole seedlings. DD plants grown in liquid medium were treated with EtOH or 5 μM DEX and were kept under light for indicated periods of time. H2O2 and O2•− were visualized by DAB and NBT staining, respectively. DAB, 3,3′-diaminobenzidine; DD, GVG-NtMEK2DD; DEX, dexamethasone; EtOH, ethanol; MPK, mitogen-activated protein kinase; NBT, nitroblue tetrazolium; ROS, reactive oxygen species. (TIF) [file pbio.2004122.s002.tif]

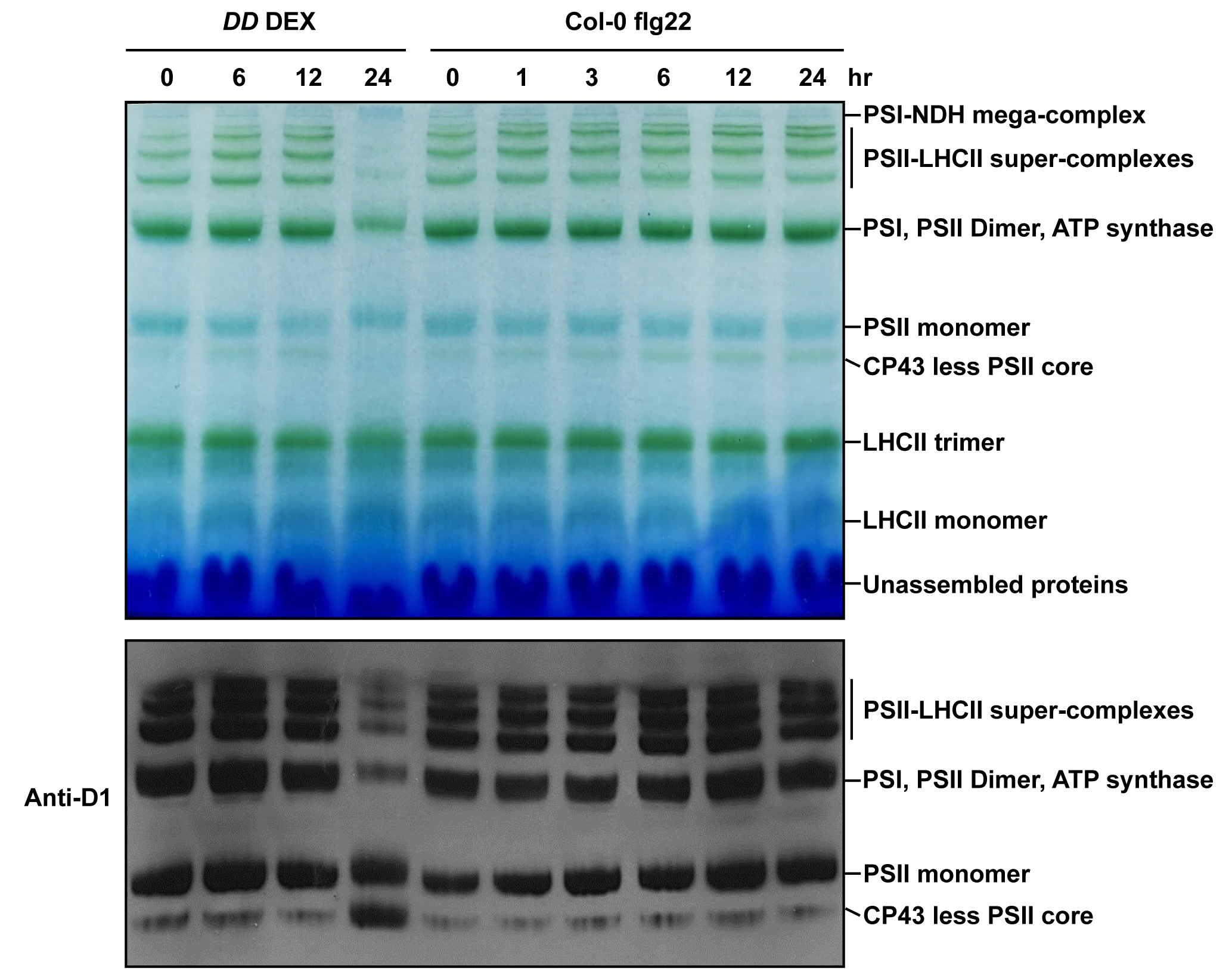

Supplement: S3 Fig — Twelve-d-old DD and Col-0 plants grown in liquid medium were treated with 5 μM DEX and 200 nM flg22 for indicated times, respectively. Thylakoid membranes were isolated and solubilized with 1% dodecyl maltoside. Samples equivalent to 8 μg of chlorophyll were loaded to a BN-PAGE. For detection of D1 abundance in different complexes, samples equivalent to 2 μg of chlorophyll were loaded to a BN-PAGE. After transferring to a PVDF membrane, anti-D1 was used to detect D1 abundance. BN-PAGE, blue native polyacrylamide gel electrophoresis; Col-0, Columbia-0; DEX, dexamethasone; flg22, a 22 amino acids flagellin fragment; MPK, mitogen-activated protein kinase; PSII, photosystem II; PVDF, polyvinylidene fluoride. (TIF) [file pbio.2004122.s003.tif]

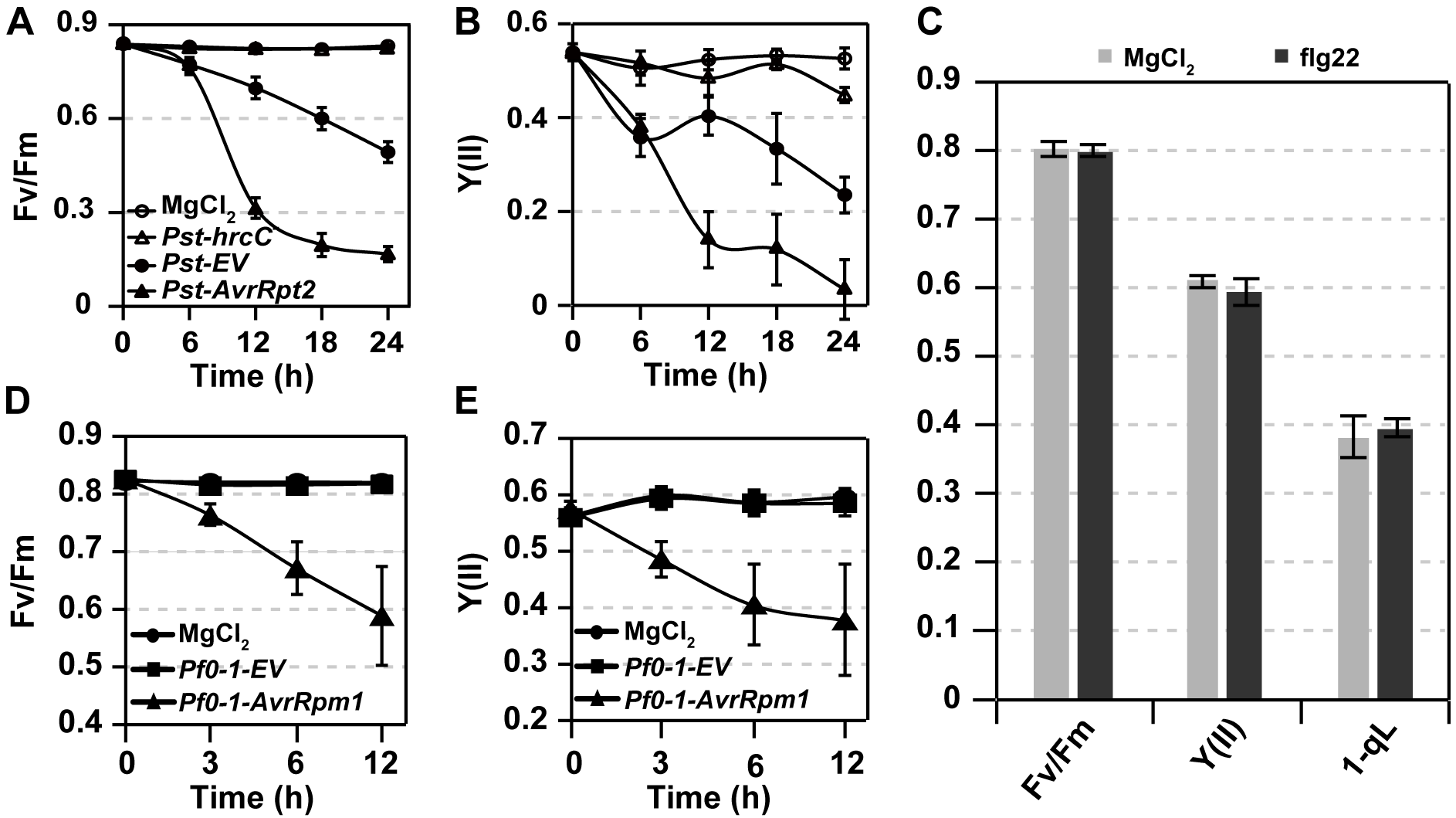

Supplement: S4 Fig — (A and B) Photosynthetic parameters are differentially affected by different Pst strains. Four-wk-old Col-0 plants were infiltrated with 10 mM MgCl2 (mock), Pst-EV, Pst-AvrRpt2, or Pst-hrcC− (OD600 = 0.2). The plants were covered with a transparent lid (100% relative humidity) and kept under light. Fv/Fm and Y(II) were measured at the indicated time. Values are means ± SD, n = 8. (C) Flg22-triggered PTI does not induce photosynthetic inhibition. Four-wk-old Col-0 plants were infiltrated with 10 mM MgCl2 (mock) or 50 nM flg22. Photosynthetic parameters were measured at 24 hpi. Values are means ± SD, n = 8. (D and E) AvrRpm1-triggered ETI also induces photosynthetic inhibition. Four-wk-old Col-0 plants were infiltrated with 10 mM MgCl2 (mock), P. fluorescens-EV (Pf0-1-EV), or P. fluorescens-AvrRpm1 (Pf0-1-AvrRpm1) (OD600 = 0.02). Fv/Fm and Y(II) were measured at indicated time points. Values are means ± SD, n = 8. The numerical values used to construct panels A–E can be found in S1 Data. AvrRpm1, avirulence effector recognized by RPM1; AvrRpt2, avirulence effector recognized by RPS2; Col-0, Columbia-0; ETI, effector-triggered immunity; EV, empty vector; flg22, a 22 amino acids flagellin fragment; hpi, hours post inoculation; hrcC−, outer membrane type III secretion protein HrcC mutant; OD, optical density; Pf0-1-AvrRpm1, P. fluorescens-AvrRpm1; Pf0-1-EV, P. fluorescens-EV; Pst, Pseudomonas syringae pv tomato; PTI, PAMP-triggered immunity. (TIF) [file pbio.2004122.s004.tif]

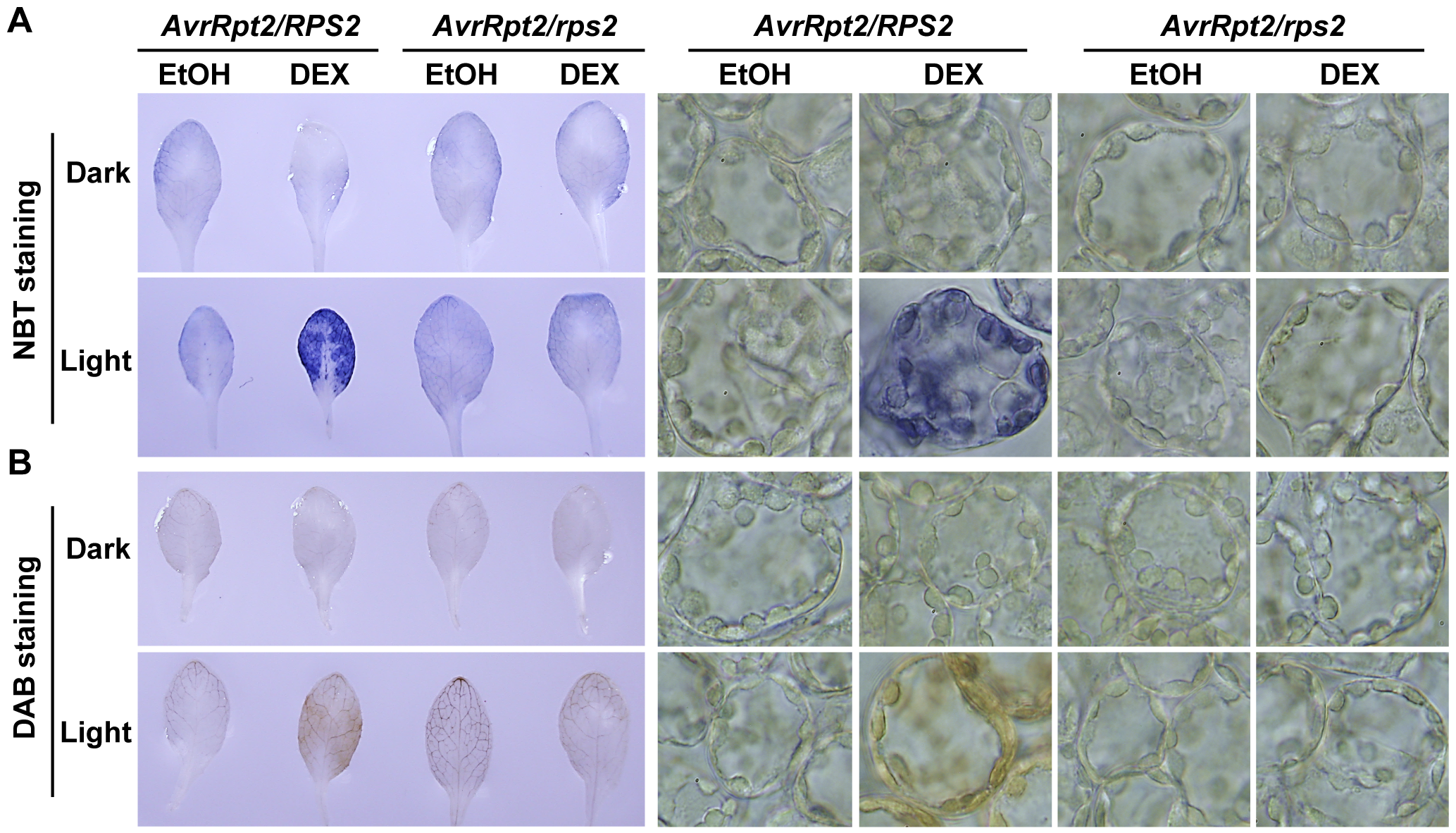

Supplement: S5 Fig — AvrRpt2-mediated ETI results in light-dependent increases in O2•− and H2O2 production in chloroplasts. Twelve-d-old GVG-AvrRpt2/RPS2 and GVG-AvrRpt2/rps2 plants were treated with DEX (5 μM) or EtOH solvent control for 8 h. O2•− and H2O2 accumulation was detected by NBT (A) and DAB (B) staining, respectively. Images of ROS accumulation at whole leaf level and subcellular level were shown. AvrRpt2, avirulence effector recognized by RPS2; DAB, 3,3′-diaminobenzidine; DEX, dexamethasone; ETI, effector-triggered immunity; EtOH, ethanol; GVG-AvrRpt2, DEX-inducible promoter-driven AvrRpt2; NBT, nitroblue tetrazolium; O2•−, superoxide; ROS, reactive oxygen species; RPS2, Resistance to Pseudomonas syringae 2. (TIF) [file pbio.2004122.s005.tif]

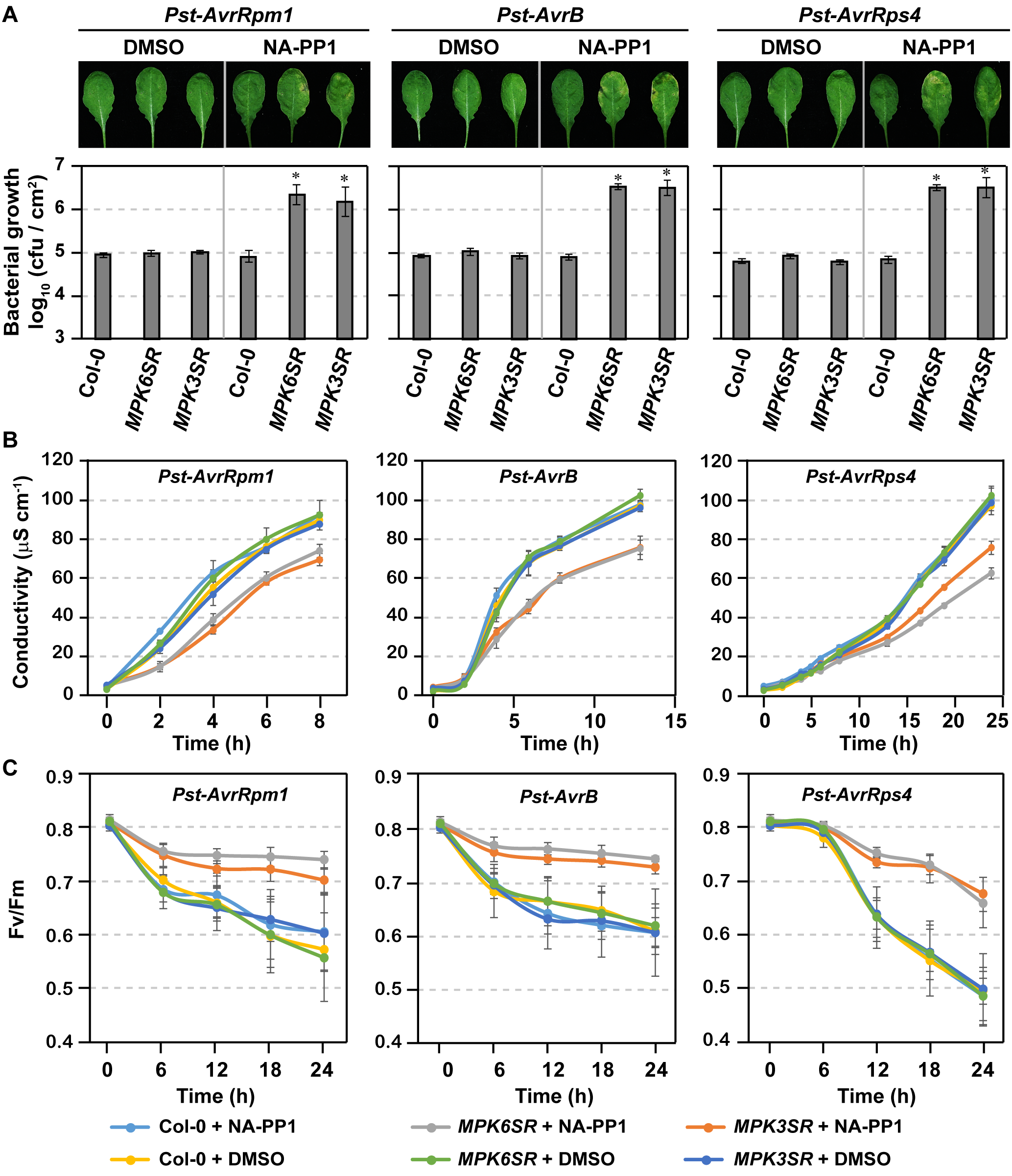

Supplement: S6 Fig — (A) Four-wk-old plants were first sprayed with DMSO or 10 μM NA-PP1 for 2 h before infiltration with Pst-AvrRpm1 (OD600 = 0.0005), Pst-AvrB (OD600 = 0.0005), or Pst-AvrRps4 (OD600 = 0.0005). NA-PP1 or DMSO was sprayed again at 1.5 dpi. Pst-AvrRpt2 growth was quantified at 3 dpi. Values are means ± SD, n = 3, *P ≤ 0.001. (B) Plants pretreated with DMSO or 10 μM NA-PP1 were infiltrated with Pst-AvrRpm1 (OD600 = 0.02), Pst-AvrB (OD600 = 0.02), or Pst-AvrRps4 (OD600 = 0.02). Soil-grown Col-0, MPK6SR, and MPK3SR plants were first spray treated with 10 μM NA-PP1 for 2 h, and leaf discs were punched and then infiltrated with Pst-AvrRpt2 (OD = 0.02) by vacuum. Leaf discs were then transferred to GC vials containing 2 μM NA-PP1 or DMSO. Ion leakage was measured as increase in conductivity. Values are means ± SD, n = 3. (C) Four-wk-old soil-grown Col-0, MPK6SR, and MPK3SR plants were first spray treated with NA-PP1 (10 μM) or DMSO solvent control for 2 h and then infiltrated with Pst-AvrRpm1 (OD = 0.02), Pst-AvrB (OD = 0.02), and Pst-AvrRps4 (OD = 0.02). Values are means ± SD, n = 8. The numerical values used to construct panels A–C can be found in S1 Data. AvrB, avirulence protein B; AvrRpm1, avirulence effector recognized by RPM1; AvrRps4, avirulence effector recognized by RPS4; CNL, coiled coil-nucleotide binding site-leucine rich repeat; Col-0, Columbia-0; ETI, effector-triggered immunity; GC, gas chromatography; MPK, mitogen-activated protein kinase; NA-PP1, 4-amino-1-tert-butyl-3-(1’-naphthyl)pyrazolo[3,4-d]pyrimidine; NLR, nucleotide-binding leucine-rich repeat; OD, optical density; Pst, Pseudomonas syringae pv tomato; TNL, toll/interleukin-1 receptor-nucleotide binding site-leucine rich repeat. (TIF) [file pbio.2004122.s006.tif]

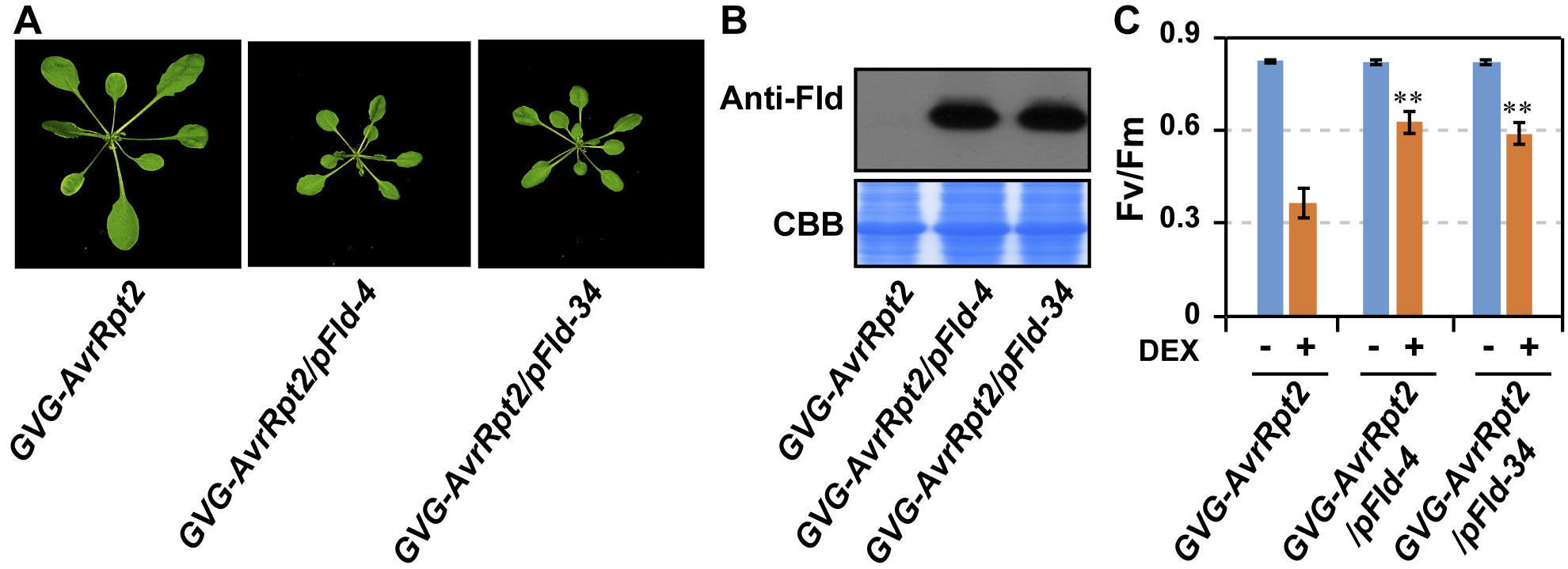

Supplement: S7 Fig — (A) Expression of pFld causes growth retardation. Four-wk-old soil-grown pFld transgenic plants in GVG-AvrRpt2 background were shown. (B) Immunoblot analysis of Fld expression in pFld transgenic plants using anti-Fld antibody. (C) AvrRpt2-induced PSII inactivation is delayed in pFld transgenic lines. Twelve-d-old GVG-AvrRpt2, GVG-AvrRpt2/pFld-4, and GVG-AvrRpt2/pFld-34 plants grown in liquid medium were treated with EtOH or 5 μM DEX. Fv/Fm was measured at 18 hpi. Values are means ± SD, n = 6, **P ≤ 0.001. The numerical values used to construct panel C can be found in S1 Data. AvrRpt2, avirulence effector recognized by RPS2; DEX, dexamethasone; EtOH, ethanol; Fld, flavodoxin; GVG-AvrRpt2, DEX-inducible promoter-driven AvrRpt2; hpi, hours post inoculation; pFld, plastid-targeted cyanobacterial flavodoxin; PSII, photosystem II. (TIF) [file pbio.2004122.s007.tif]

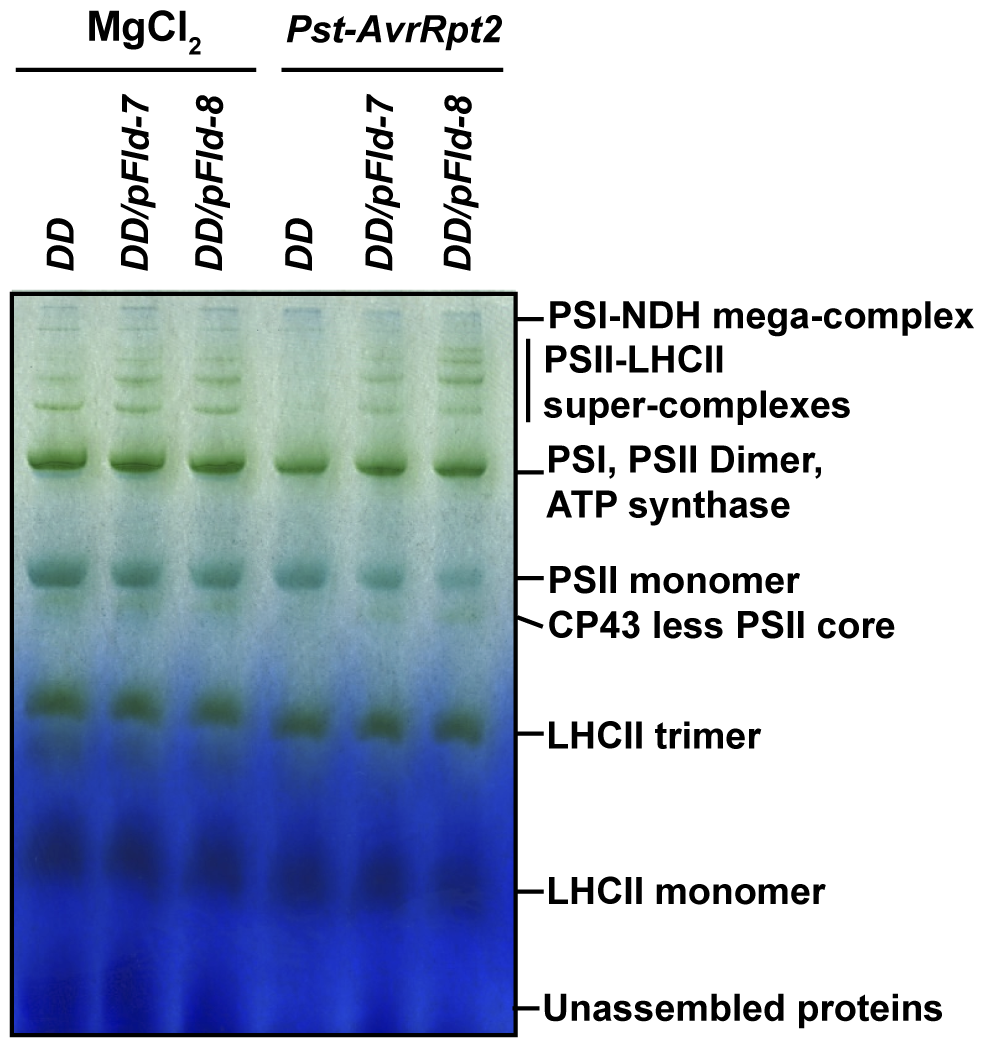

Supplement: S8 Fig — Twelve-d-old DD and DD pFld plants grown in liquid medium were treated with Pst-AvrRpt2 (OD = 0.02); disassembly of photosynthetic complexes at 24 hpi was visualized with BN-PAGE. Thylakoid membranes were isolated and solubilized with 1% dodecyl maltoside. Samples equivalent to 8 μg of chlorophyll were loaded to a blue native polyacrylamide gel (BN-PAGE). AvrRpt2, avirulence effector recognized by RPS2; BN-PAGE, blue native polyacrylamide gel electrophoresis; DD, GVG-NtMEK2DD; hpi, hours post inoculation; OD, optical density; pFLD; plastid-targeted cyanobacterial flavodoxin; PSII, photosystem II; Pst, Pseudomonas syringae pv tomato. (TIF) [file pbio.2004122.s008.tif]

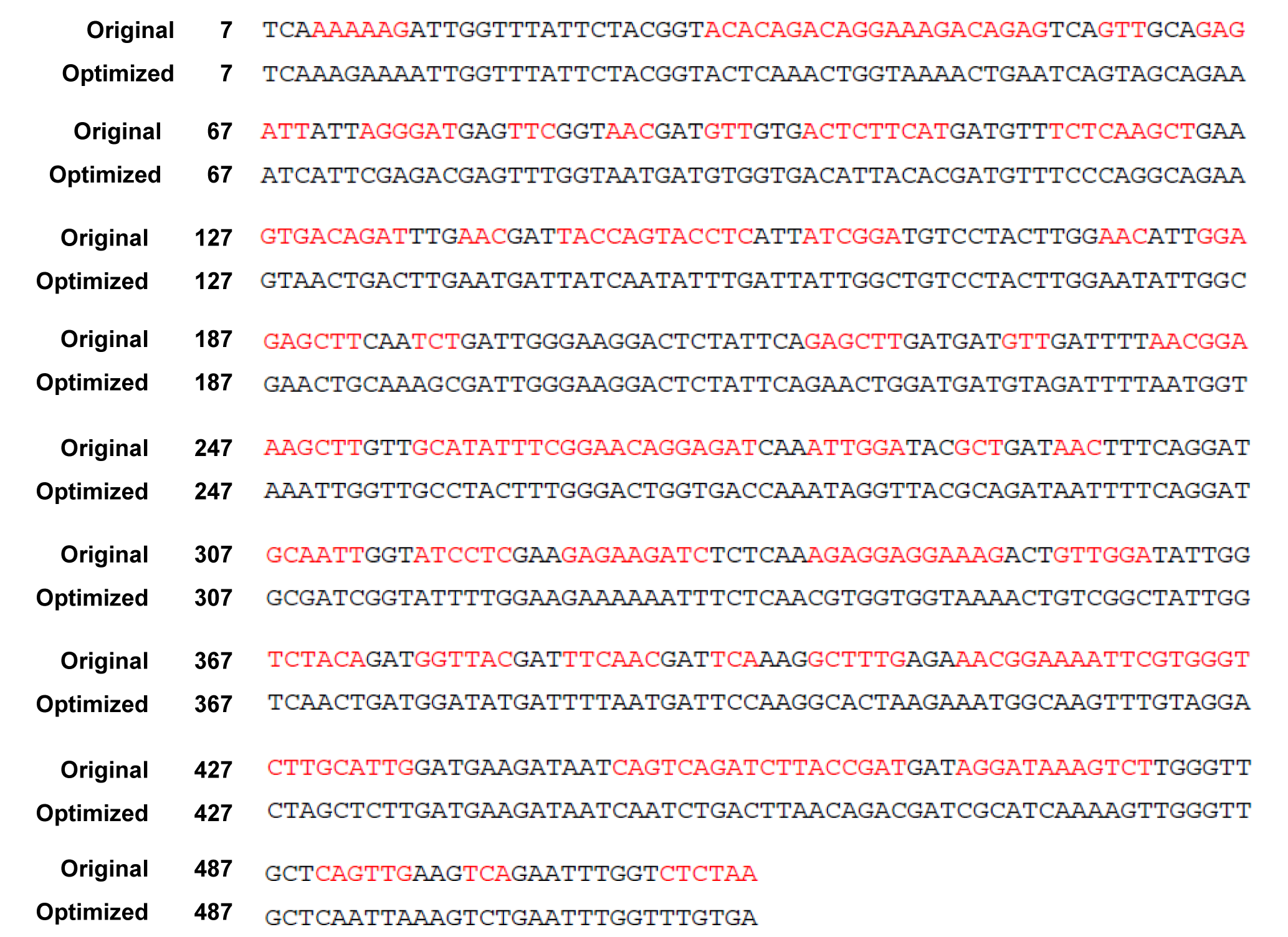

Supplement: S9 Fig — The coding sequence of Fld from cyanobacterium Anabaena sp. PCC 7119 was optimized using OptimumGene algorithm (Genscript, http://www.genscript.com). Fld, flavodoxin. (TIF) [file pbio.2004122.s009.tif]
